# Oxygen isotope composition of Mesoproterozoic (~1360 Ma) seawater constrained by clumped isotopes of North China limestones

**DOI:** 10.1126/sciadv.adu6693

**Published:** 2025-10-17

**Authors:** Pingping Li, Fang Hao, Shijie He, Haobo Chai, Yongfei Jiao, Yaxuan Gao, Huayao Zou

**Affiliations:** ^1^ State Key Laboratory of Petroleum Resources and Engineering, China University of Petroleum (Beijing), Beijing, 102249, China.; ^2^ College of Geosciences, China University of Petroleum (Beijing), Beijing, 102249, China.; ^3^ Carbonate Research Center, China University of Petroleum (Beijing), Beijing, 102249, China.; ^4^ State Key Laboratory of Deep Oil and Gas, China University of Petroleum (East China), Qingdao, Shandong, 266580, China.

## Abstract

The oxygen isotope composition (δ^18^O) of Phanerozoic seawater has been widely investigated, but the δ^18^O values of Precambrian seawater remain poorly constrained, with ongoing debate over whether they were substantially lower than those of Phanerozoic seawater. To address this question, we analyzed the clumped isotopes of limestones from the North China Craton to reconstruct Mesoproterozoic seawater temperature and δ^18^O values. Our results indicate that the Mesoproterozoic seawater had a temperature of 26.9° ± 0.4°C and a δ^18^O value of −6.3 ± 0.2 per mil (relative to standard mean ocean water). This δ^18^O estimate aligns with previous inferences from geochemical modeling, marine iron oxides, and oxygen isotope ensembles, supporting the hypothesis that Mesoproterozoic seawater was isotopically lighter than its Phanerozoic counterpart. These findings provide insights into the Earth’s paleoclimate and the evolution of seawater composition during the Mesoproterozoic era.

## Teaser

The δ^18^O value of Mesoproterozoic seawater (−6.3 ± 0.2‰) is substantially lower than that of modern seawater.

## INTRODUCTION

The δ^18^O composition of seawater is primarily influenced by submarine alteration and continental weathering (*1*–*3*). The secular evolution of seawater δ^18^O provides critical insights into Earth’s surface environments and the history of oceanic crust hydrothermal alteration (*4*, *5*). Furthermore, the δ^18^O signature of paleo-seawater serves as a fundamental parameter for reconstructing carbonate precipitation temperature and understanding related diagenetic processes, including marine dolomite formation (*6*–*8*). Current scientific inquiry is mainly focused on characterizing seawater δ^18^O evolution (*1*–*3*, *5*, *9*–*11*) with two contrasting views: (i) a stable seawater δ^18^O value throughout Earth’s history [0 ± 2 per mil (‰), e.g., (*12*)] versus (ii) a progressive ~10‰ increase since the Archean [e.g., (*3*, *4*)]. While the δ^18^O evolution of Phanerozoic seawater has been systematically reconstructed using fossil calcitic and phosphatic shells (*5*, *9*), Precambrian seawater δ^18^O remains poorly constrained, with only model-based estimates available for the past 3.4 billion years (Ga) (*3*). Recent studies have provided limited seawater δ^18^O values derived from iron oxide and silica samples for 1.5 to 2 Ga (*10*, *11*). Although these studies consistently support a long-term increase in seawater δ^18^O values (*3*, *5*, *10*), additional geological records are urgently required to better constrain the Precambrian seawater δ^18^O values, particularly during the Mesoproterozoic era (1.85 to 0.85 Ga). This critical period in Earth’s history, often referred to as the “boring billion” (*13*) due to a slow diversification relative to the following Cambrian explosion, nonetheless represents a crucial period that established the environmental conditions for the eventual emergence of complex life.

The reconstruction of ancient seawater δ^18^O values using conventional oxygen isotope thermometers requires knowledge of both the minerals δ^18^O values and their formation temperatures (*10*). However, the temperatures at which marine authigenic minerals (such as carbonate minerals or shells) are deposited are often unknown (*14*). In current studies of seawater δ^18^O evolution, these temperatures are derived on the basis of empirical temperature calibrations of isotope fractionation factors between water and carbonate minerals [e.g., (*15*)], assuming a fitted baseline for the evolution of seawater δ^18^O (*5*). Postdepositional alteration further complicates these methods, as burial diagenesis and recrystallization can substantially modify original mineral δ^18^O signatures (*8*, *16*). Carbonate clumped isotope analyses can directly determine the formation temperatures of carbonate minerals without requiring a priori knowledge of fluid δ^18^O values (*14*, *17*). This approach relies on the assumption that the minerals have attained thermodynamic equilibrium—a condition that can be independently assessed using dual clumped isotopes [Δ_47_ and Δ_48_ (*18*–*21*)]. When combined with conventional oxygen isotope thermometry, carbonate clumped isotope analyses allow for robust reconstruction of parent fluid δ^18^O compositions. This integrated approach has emerged as a particularly powerful method for determining paleo-seawater δ^18^O values, as demonstrated by multiple recent studies (*22*–*24*).

**Fig. 1.**
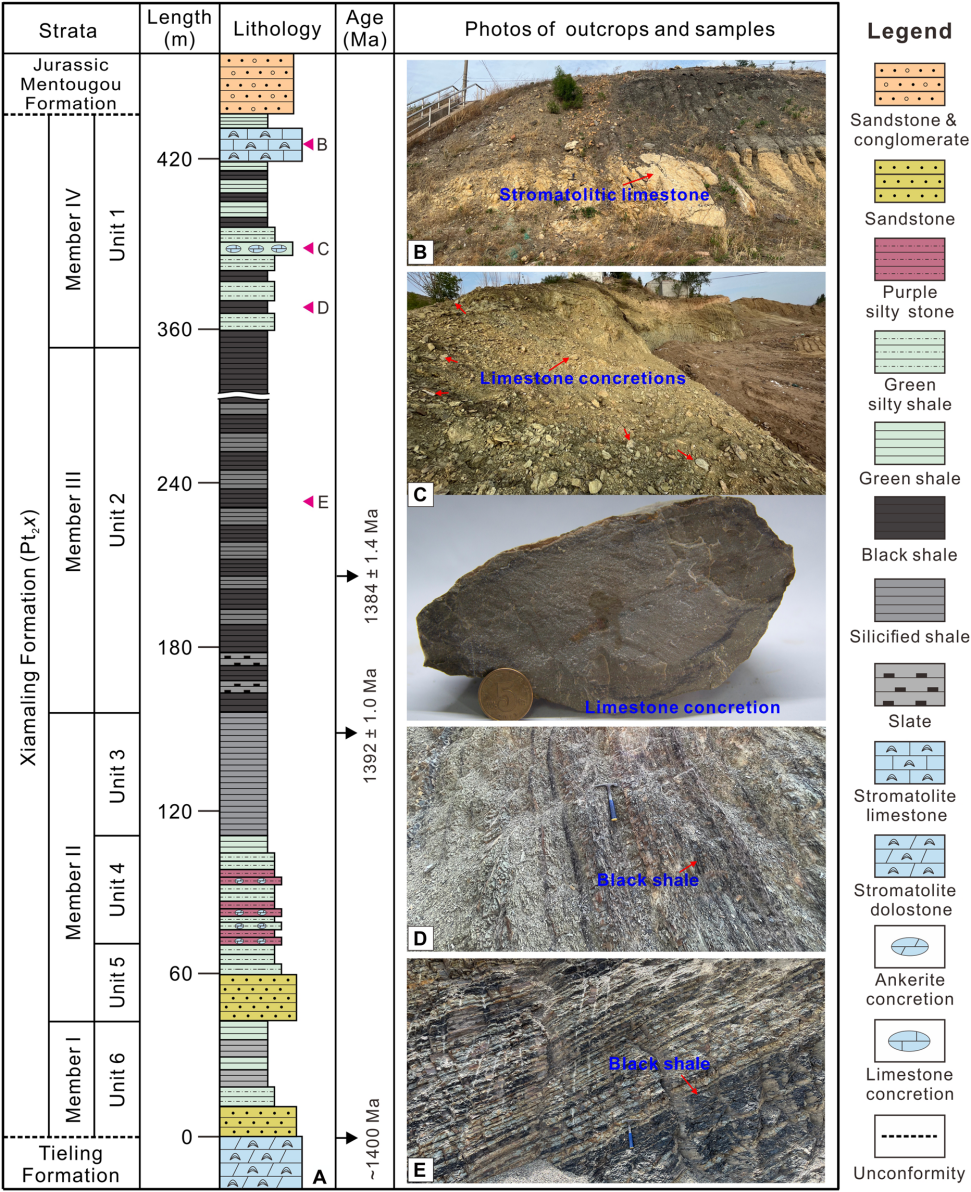
General lithological column and outcrop photos. (**A**) Lithological column of the Mesoproterozoic Tieling, Xiamaling, and Jurassic Mentougou formations in the Xiahuanyuan area [modified from (*25*, *78*)]. (**B**) Stromatolitic limestone and (**C**) limestone concretion samples were collected from the upper and middle parts of unit 1, and (**D** and **E**) the two shale samples were collected from units 1 and 2. Reprinted from (*25*), copyright (2020), with permission from Elsevier. Reprinted from (*78*), copyright (2017), with permission from Yale University-AJS.

The upper Mesoproterozoic Xiamaling Formation (Pt_2_*x*) along the northern margin of the North China Craton (NCC) contains limestone concretions and stromatolitic limestone (Fig. 1 and figs. S1 and S2) (*25*, *26*). Organic maturity indicators of the Pt_2_*x* shale suggest a maximum burial temperature below 90°C (*27*), representing an exceptionally mild burial history for Mesoproterozoic strata. The limited thermal exposure strongly suggests that these carbonate samples have avoided substantial high-temperature diagenetic alteration that can alter oxygen and clumped isotope compositions. Consequently, these samples are particularly valuable for preserving primary isotopic compositions acquired at or near the ancient Earth surface. This article aims to (i) determine the Mesoproterozoic seawater temperature using clumped isotopes of the Pt_2_*x* limestones from NCC, (ii) reconstruct the Mesoproterozoic seawater δ^18^O value by combining seawater temperature with δ^18^O values of limestone samples, and (iii) assess the potential for substantially negative seawater δ^18^O values during the Mesoproterozoic through comparison with existing model predictions and geochemical proxies. Our findings provide crucial constraints on Mesoproterozoic seawater δ^18^O values and contribute to resolving the secular evolution of seawater δ^18^O values during this period in Earth history.

### Geological background

The Xuanlong depression, located within the Yanliao Faulted-Depression Zone at the northern margin of the NCC (fig. S1), contains Mesoproterozoic strata comprising the Tieling Formation (Pt_2_*t*) overlain by the Xiamaling Formation (Pt_2_*x*) (fig. S2). The underlying Pt_2_*t* is characterized by thick dolostone sequences capped by a distinct weathered crust (*28*), while the Pt_2_*x* was deposited in a back-arc basin environment associated with oceanic crust subduction (*29*). Within the Xuanlong depression, the Pt_2_*x* exhibits a well-defined stratigraphic succession divided into four members or six units from bottom to top (Fig. 1) (*25*, *30*). Member I (unit 6) consists of sandstones and sandy shales; member II is composed of sandstones and purple mudstones (units 5 and 4) transitioning upward into gray siliceous shales (unit 3); member III (unit 2) is predominantly black shales; and member IV (unit 1) features gray-green shales with mid-section limestone concretions overlain by stromatolitic limestone. The succession is unconformably overlain by Jurassic Mentougou Formation (J_1_*m*) or Tiaojishan Formation (J_2_*t*). Geochronological constraints indicate the Pt_2_*t*/Pt_2_*x* boundary at 1400 million years (Ma) (*31*), with zircon ages of 1392.2 ± 1.0 Ma from member II bentonite layers (*27*) and 1384 ± 1.4 Ma/1366 ± 9 Ma from member III tuff layers (*27*, *32*).

The Pt_2_*x* has undergone multiple phases of uplift (fig. S2) and is now exposed at the surface in the Xuanlong depression and surrounding areas (fig. S3). Around 1100 Ma, the Weixian uplift triggered denudation of the Pt_2_*x* (*33*), followed by Neoproterozoic sedimentation (1100 to 800 Ma) associated with the breakup of the Rodinia supercontinent (*34*). The Jixian movement (800 to 541 Ma) further uplifted the NCC, resulting in the closure of the Faulted-Depression (*35*). During the Paleozoic (541 to 455 Ma), the northern NCC accumulated epicontinental marine carbonates (*36*), before the Caledonian orogeny (455 to 320 Ma) induced regional uplift and erosion. Sedimentation resumed from the Middle Carboniferous to Middle Triassic, only to be interrupted again during the Late Triassic due to Pangaea assembly, which subjected the NCC to intense lateral compression of the NCC, uplift, and denudation (*37*). Subsequent Jurassic deposition was later modified by the Late Jurassic intracontinental compression, leading to renewed uplift and erosion across the northern NCC (*38*). As a result, the Xuanlong depression predominantly preserves only Lower or Middle Jurassic strata overlying the Pt_2_*x*, whereas other parts of North China retain more complete Neoproterozoic, Paleozoic, and Mesozoic successions between Jurassic and Mesoproterozoic units.

## RESULTS AND DISCUSSION

### Clumped isotopes and formation temperature of limestone

The formation temperatures estimated through traditional oxygen thermometry require assuming a known δ^18^O value for the water from which carbonates precipitate or recrystallize. However, temperature estimates derived from empirical calibrations (*5*) exhibit higher uncertainty due to the scarcity of δ^18^O data available for Precambrian samples. In contrast, carbonate clumped isotopes provide a more direct approach for determining the formation temperatures of carbonate minerals, independent of fluid δ^18^O composition (*14*). Since the initial measurement of Δ_47_ (the measured abundance of mass 47 isotopologs relative to their expected abundance under stochastic distribution) in 2003, multiple temperature calibration equations gained wide acceptance [e.g., (*39*–*41*)]. Most discrepancies between different temperature calibrations are being resolved within the InterCarb-Carbon Dioxide Equilibrium Scale (I-CDES) reference frame (*42*). The subsequent development of Δ_48_ measurements in 2019 (*43*) enabled the establishment of a temperature calibration equation and an independent thermometer (*44*). Together, dual clumped isotopes (Δ_47_ and Δ_48_) provide a powerful tool for assessing the clumped isotope thermodynamic equilibrium in carbonate materials (*18*–*21*), offering critical insights into carbonate formation and diagenetic histories.

**Fig. 2.**
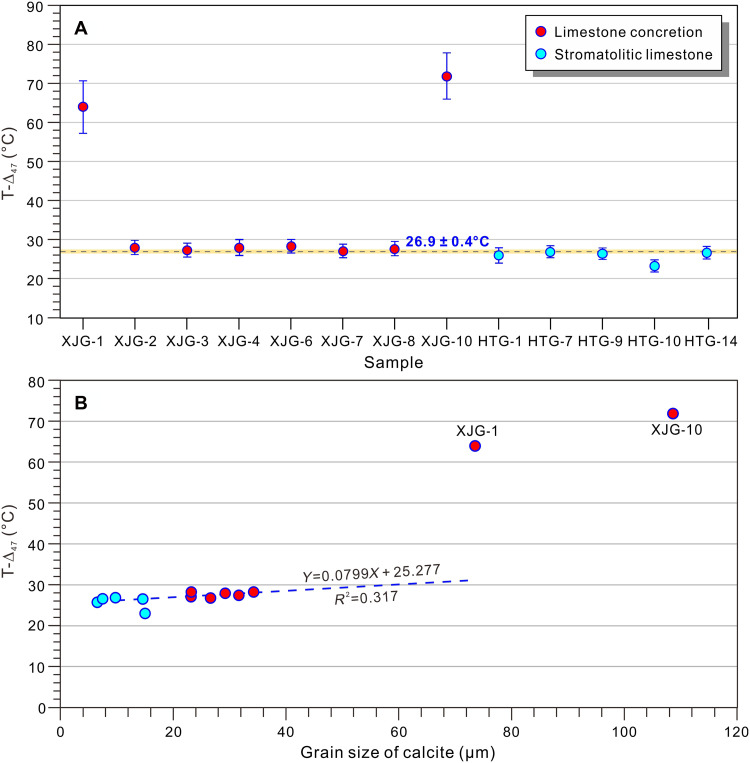
Clumped isotope temperatures (T-Δ_47_) of limestones. (**A**) Detailed T-Δ_47_ values for each sample. Gray dashed line represents the average T-Δ_47_ value (26.9°C) excluding samples XJG-1 and XJG-10. Yellow shading represents the average margin of error for all samples (±0.4°C). (**B**) Variations in clumped isotope values (T-Δ_47_) with grain size of calcite. Grain size is mostly temperature independent from 5 to 40 μm but shows a significant increase—likely during recrystallization at elevated burial temperatures above 60°C.

Preservation of primary carbonate mineralogy is essential for accurately determining initial formation temperatures and reconstructing the properties of original diagenetic fluids (*45*). Our x-ray diffraction (XRD) analyses demonstrate that both the limestone concretions and stromatolitic limestones are predominantly composed of calcite (80 to 95%), with subordinate quartz (5 to 19%) and minor amounts of dolomite and clay minerals (table S1). According to the previous classification of limestones (*46*), the calcite grains in stromatolitic limestones and limestone concretions are very fine- to fine-crystalline (microcrystalline), with mean grain sizes of 10.7 and 27.9 μm, respectively (table S1). These sizes closely resemble those of primary precipitated calcite grains, which are ~10 μm. The T-Δ_47_ (Δ_47_-calibrated temperatures) of the very fine- to fine-crystalline limestone (23.3° to 28.4°C; Table 1) are substantially lower than those obtained from two coarse-crystalline limestone concretion samples (XJG-1 and XJG-10, 64.0° to 71.9°C; Fig. 2A). Three lines of evidence support the preservation of primary signatures (no substantial recrystallization or diagenetic alteration) in the very fine- to fine-crystalline samples: (i) the absence of a systematic relationship between calcite grain size and T-Δ_47_ (Fig. 2B), (ii) lack of cathodoluminescence (fig. S4), and (iii) grain sizes consistent with primary precipitates. In contrast, the two coarse-crystalline limestone concretions (>50 μm) exhibit bright cathodoluminescence (fig. S4), indicating substantial late-stage recrystallization and thermal resetting of their isotopic signatures.

**Table 1.** δ^13^C, δ^18^O, Δ_47_, T-Δ_47_, and Δ_48_ values of limestones. The numbers in parentheses represent sample replicates. The uncertainties of δ^13^C, δ^18^O, Δ_47_, and Δ_48_ are reported as SEs, while the uncertainty in T-Δ_47_ represents the average of the upper and lower bounds derived from propagating the Δ_47_ uncertainty through the temperature calibration equation.

Sample ID	δ^13^C (‰VPDB)	δ^18^O (‰VPDB)	Δ_47_ (I-CDES90, ‰)	T_Δ47_ (°C)	Δ_48_ (I-CDES90, ‰)
XJG-1	−0.294 ± 0.011	−8.323 ± 0.008	0.496 ± 0.014(2)	64.0 ± 6.7	0.205 ± 0.017(2)
XJG-2	0.168 ± 0.005	−7.988 ± 0.005	0.585 ± 0.005(6)	28.0 ± 1.8	0.241 ± 0.013(2)
XJG-3	0.681 ± 0.005	−8.779 ± 0.003	0.587 ± 0.005(5)	27.3 ± 1.8	–
XJG-4	−0.203 ± 0.005	−8.125 ± 0.005	0.585 ± 0.006(5)	28.0 ± 2.0	–
XJG-6	−1.997 ± 0.007	−9.761 ± 0.007	0.584 ± 0.005(6)	28.4 ± 1.8	0.239 ± 0.011(5)
XJG-7	−0.234 ± 0.007	−9.253 ± 0.005	0.588 ± 0.005(6)	27.1 ± 1.7	0.242 ± 0.011(5)
XJG-8	−0.253 ± 0.004	−9.438 ± 0.005	0.586 ± 0.005(6)	27.7 ± 1.8	–
XJG-10	0.493 ± 0.008	−7.605 ± 0.005	0.482 ± 0.012(3)	71.9 ± 5.9	0.196 ± 0.020(2)
HTG-1	0.966 ± 0.006	−7.603 ± 0.006	0.591 ± 0.006(4)	26.0 ± 2.0	0.236 ± 0.012(3)
HTG-7	−0.074 ± 0.004	−8.281 ± 0.004	0.588 ± 0.004(9)	26.9 ± 1.4	0.248 ± 0.008(5)
HTG-9	0.655 ± 0.005	−7.842 ± 0.005	0.590 ± 0.004(6)	26.3 ± 1.4	–
HTG-10	0.959 ± 0.005	−7.382 ± 0.004	0.599 ± 0.004(6)	23.3 ± 1.3	0.244 ± 0.011(6)
HTG-14	0.798 ± 0.005	−7.778 ± 0.005	0.589 ± 0.005(6)	26.7 ± 1.6	–

The likely diagenetic alteration of limestone was the aragonite-to-calcite transition during neomorphism in this study. While the Mg/Ca ratio of the Mesoproterozoic seawater remains less constrained than in Phanerozoic seawater, two evidence indicates the Mesoproterozoic ocean maintained an “aragonite sea” geochemical regime, characterized by the following: (i) Carbonate-evaporite succession from 1.2 Ga suggested that Mesoproterozoic seawater had an Mg^2+^ concentration substantially greater than modern seawater (*47*) and (ii) globally extensive, thick successions (>3000 m in the NCC) of syndepositional dolostones (*48*, *49*), providing further evidence for elevated Mg^2+^ concentrations in Mesoproterozoic seawater. These conditions would have favored primary precipitation of high-Mg calcite (aragonite). The aragonite-to-calcite transition occurred on the seafloor, and Δ_47_ values showed no substantial variation during this transition (*50*). Except for in the two coarsely crystalline limestone samples, the T-Δ_47_ values of the other limestone samples remained relatively stable (Fig. 2A). Although there is rough positive relation between the δ^18^O and δ^13^C values (fig. S5A), there is nonlinear relationship between the δ^18^O and the T-Δ_47_ values of limestone samples (fig. S5B), suggesting that no substantial diagenetic alteration occurred in these limestones as the δ^18^O values gradually become more depleted during diagenetic alteration. In summary, our clumped isotope data reveal distinct diagenetic histories: Most limestone samples maintain consistently low Δ_47_ temperatures (mean, 26.9° ± 0.4°C), indicating negligible burial alteration (*22*), while two anomalous samples (XJG-1 and XJG-10) display elevated temperatures characteristic of recrystallization. Thus, the limestones with low T-Δ_47_ values preserved the surface or near-surface isotope compositions.

**Fig. 3.**
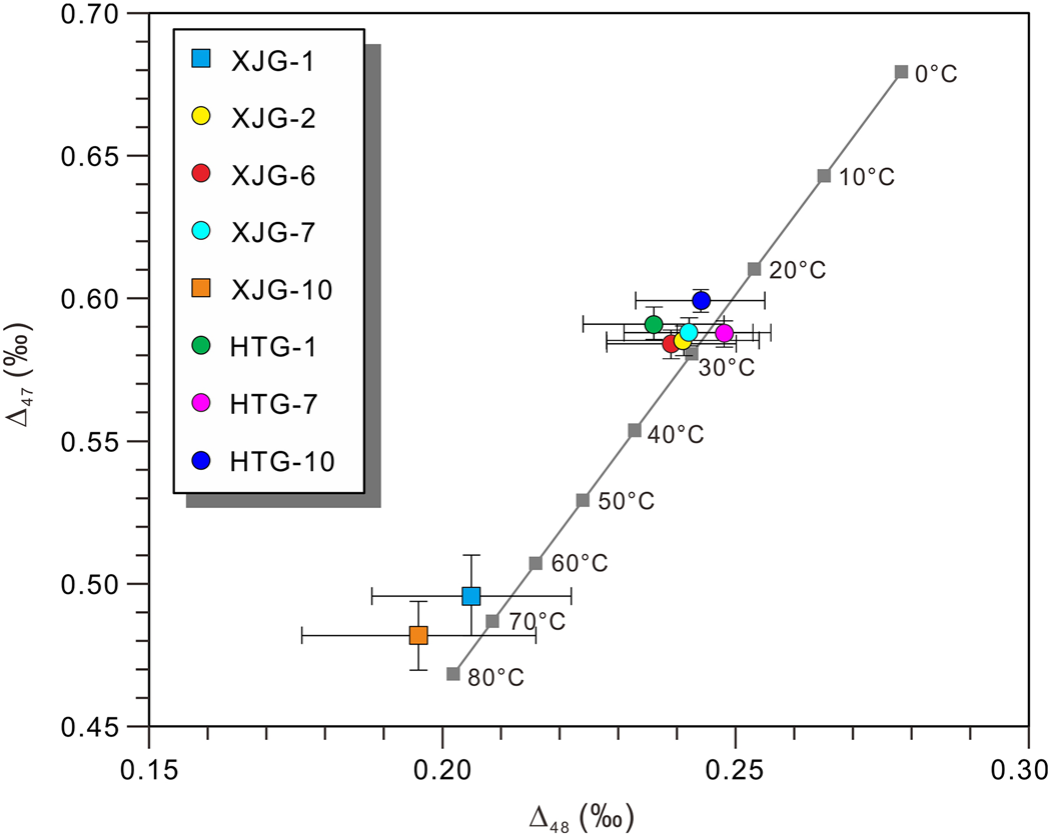
Plot of Δ_47_ versus Δ_48_ values of limestones. All samples are plotted near the gray line of clumped isotope equilibrium, which was obtained using the empirical relationship previously described in (*18*): $\begin{array}{c} \Delta_{47\left(\text{CDES}90\right)}=-0.4771+9.102\times \Delta_{48\left(\text{CDES}90\right)}- \end{array}$ $\begin{array}{c} 31.709\times \Delta_{48\left(\text{CDES}90\right)}^{2}+65.561\times \Delta_{48\left(\text{CDES}90\right)}^{3}-54.560\times \Delta_{48\left(\text{CDES}90\right)}^{4} \end{array}$

The accurate determination of carbonate formation temperatures using clumped isotope thermometry (T-Δ_47_ or T-Δ_48_) fundamentally requires isotopic equilibrium during mineral precipitation. The equilibrium of clumped isotopes in carbonate minerals is achieved by the balance between CO_2_ hydration, hydroxylation, and CO_2_ degassing (*51*). However, certain carbonate systems, including cave-filled carbonates (e.g., speleothems), biogenic calcites (e.g., corals), and microbially involved carbonates, often exhibit kinetic isotope effects, leading to deviations from clumped isotope equilibrium (*18*, *19*). Consequently, the clumped isotope temperatures (T-Δ_47_ or T-Δ_48_) may not accurately reflect the true initial formation temperatures. The development of dual-clumped isotope analysis (Δ_47_ and Δ_48_) provides a robust method for verifying equilibrium conditions (*18*–*21*). Thermodynamic principles predict that equilibrium samples should plot along a theoretical line where T-Δ_47_ equals T-Δ_48_ across all temperatures. T-Δ_47_ and T-Δ_48_ values of carbonate samples that fall on or near the theoretical equilibrium line indicate that the samples have reached clumped isotope equilibrium. Conversely, deviations from this line suggest clumped isotope disequilibrium. The Δ_47_ and Δ_48_ values of our limestone samples consistently fall on or near this equilibrium line (Fig. 3), indicating that they achieved complete clumped isotope equilibrium. This validation confirms that the derived T-Δ_47_ values faithfully record the original precipitation temperatures of these carbonates.

**Fig. 4.**
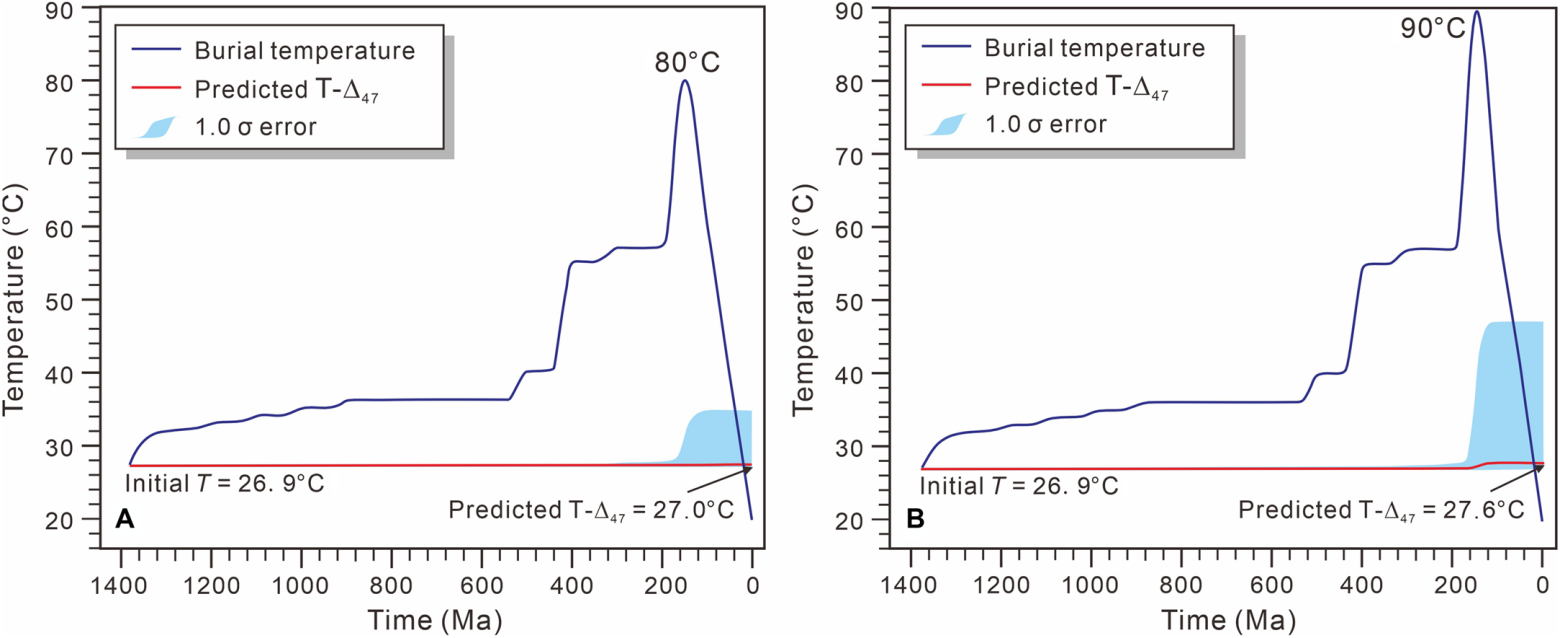
Evolution of clumped isotope temperatures (T-Δ_47_) for limestones. The final predicted T-Δ_47_ values are (**A**) 27.0°C and (**B**) 27.6°C, respectively, assuming an initial temperature of 26.9°C, and the maximum burial temperature reached 80° and 90°C at 100 Ma before decreasing to 20°C during the late uplift stage.

Last, the clumped isotope signatures (e.g., Δ_47_ and Δ_48_) of carbonate minerals can be altered during deep burial through solid-state reordering (exchange of C─O bonds) at elevated temperatures (*52*, *53*), resulting in the clumped isotope temperatures (T-Δ_47_ or T-Δ_48_) no longer represent the original formation temperatures. While the reordering mechanisms and kinetic models of Δ_47_ have been extensively characterized (*53*, *54*), the behavior of Δ_48_ under similar conditions remains unexplored. According to published kinetic models of reordering (*53*, *54*), calcite experiences partial solid-state reordering (progressive increase in T-Δ_47_ toward but not reaching the ambient temperature) above 100°C and full solid-state reordering (with a T-Δ_47_ value approaching the ambient temperature) above 175° to 200°C. Multiple evidences suggest that solid-state reordering did not occur in the Pt_2_*x* limestones. The microscopic components of organic matter in Pt_2_*x* shales include vitrinite-like macerals, lamalginites, and solid bitumen (fig. S6). The calculated equivalent vitrinite reflectance (*R*_o_) values for the two Pt_2_*x* shales are 0.45 to 0.55% (with an average value of 0.5%). On the basis of the organic matter maturity model (Easy*R*_o_ model) (*55*), the corresponding maximum temperatures are to be 80° to 90°C (fig. S7), which aligns with previous research using pyrolysis parameters for the Pt_2_*x* shale (*27*). The relatively low burial temperature (<100°C) suggests that limestones did not undergo solid-state isotopic reordering. In addition, on the basis of a kinetic model of solid-state reordering (*53*), the simulated results indicate that T-Δ_47_ values increased only by 0.1° and 0.7°C for the maximum burial temperatures of 80° and 90°C (Fig. 4), respectively. These minor increments in T-Δ_47_ values fall entirely within the measurement error. Therefore, the studied Pt_2_*x* limestones preserve primary clumped isotope signatures, unaffected by solid-state reordering processes.

**Fig. 5.**
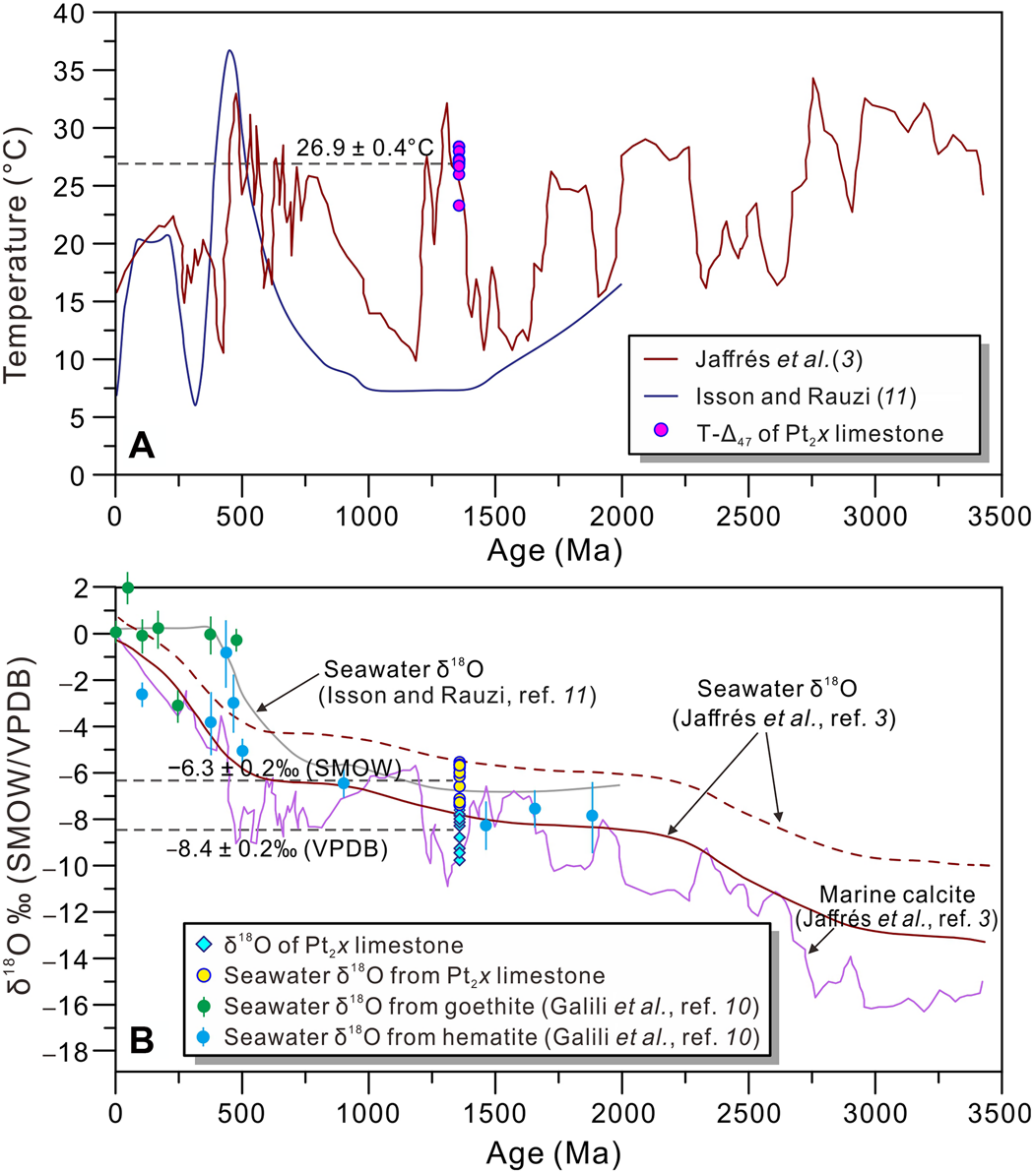
Temperature and δ^18^O evolution of seawater. (**A**) Temperature and (**B**) seawater δ^18^O determined by clumped isotope analyses compared with results from (*3*, *10*, *11*). The seawater temperature (26.9° ± 0.4°C, A) and δ^18^O values of limestones (−6.3 ± 0.2‰, B) obtained in this study agree with model predictions (*3*). Furthermore, the reconstructed Mesoproterozoic seawater δ^18^O values in this study align with both model estimates (*3*, *11*) and goethite/hematite-based reconstructions (*10*). The solid and dashed brown lines denote the modeled seawater δ^18^O evolution using standardized and relatively lower weathering and upper crust δ^18^O fractionation coefficients, respectively (B). The reconstruction of seawater δ^18^O values from goethite and hematite assumes a constant temperature evolution model (15° ± 10°C), consistent with the Mesoproterozoic temperature range derived in this study.

In summary, the limestone samples do not appear to have undergone diagenetic alteration or experience solid-state reordering and achieved clumped isotope equilibrium. Therefore, the T-Δ_47_ values can confidently represent the initial formation temperatures of the limestones. The T-Δ_47_ values provided by two different laboratories [Institute of Geology and Geophysics (IGG) and Clumped Isotopes Laboratory (CIL)] are highly consistent (table S2), with T-Δ_47_ values ranging from 23.3° ± 1.3° to 28.4° ± 1.8°C (Table 1, with an average of 26.9° ± 0.4°C; Fig. 2A). This suggests that the temperatures of Mesoproterozoic seawater were relatively warmer than those of modern seawater but did not reach the reported 52°C at 1300 Ma (*56*). The temperatures determined for Mesoproterozoic seawater at the northern margin of the NCC are consistent with the temperature based on silicon isotopes in chert [20° to 40°C; (*57*)] and with a published temperature evolution model (Fig. 5A) (*3*), further validating the reliability of the Mesoproterozoic seawater temperatures obtained in this study.

### Oxygen isotope of Mesoproterozoic seawater and its constrain on paleoclimate

In addition to seawater temperature, the reconstruction of seawater δ^18^O values requires that the δ^18^O values of carbonate minerals remains unaltered by late-stage burial processes. Three independent lines of evidences support the preservation of primary δ^18^O signatures in the studied limestone samples. First, the T-Δ_47_ of limestone samples (23.3° to 28.4°C) are relatively cold and exhibit low variability (±0.4°C), a characteristic that rules out substantial diagenetic alteration. Second, the aragonite-to-calcite transition typically results in only minor oxygen isotope fractionation [enrichment of 0.5 to 0.8‰; (*58*, *59*)], and studies of modern marine mollusk shells (formed at 14° to 29°C) show a δ^18^O enrichment of merely ~0.4‰ during the transition (*60*). The δ^18^O values of the Pt_2_*x* limestone samples [−9.8 to −7.3‰, Vienna Pee Dee belemnite (VPDB); Table 1] closely match those reported from coeval ~1360 Ma strata [−9.5 to −5.2‰; (*3*)], and the mean δ^18^O value (−8.4‰, VPDB) aligns well with model-predicted δ^18^O values for coeval calcite (−9.3‰, VPDB; Fig. 5B). Collectively, these evidences strongly suggest that the δ^18^O data obtained from our limestone samples reliably reflect the oxygen isotope composition of Mesoproterozoic seawater.

On the basis of the reconstructed Mesoproterozoic seawater temperature and δ^18^O values derived from well-preserved limestone, combined with constraints from the oxygen isotope thermometer (*58*), we estimate that the Mesoproterozoic seawater had δ^18^O values ranging from −7.4 to −5.6‰, with a mean value of −6.3 ± 0.2% [standard mean ocean water (SMOW); fig. S8]. These estimates are consistent with model-predicted δ^18^O values for contemporaneous seawater (−8.0 to −5.5‰, SMOW) (*3*), calculated using both the original and relatively lower isotopic fractionation coefficients for H_2_O uptake in the upper crust and silicate weathering (Fig. 5B). Independent estimates from coeval (1.46 Ga) marine iron oxides yield δ^18^O values of −8.4 to −7.0‰ (SMOW) (*10*), while oxygen isotope ensemble analyses suggest a value of approximately −7.5‰ (SMOW) (*11*). Although minor discrepancies exist among these datasets, our δ^18^O estimates—anchored by precise seawater temperatures determinations—are consistently and substantially lower than those of Phanerozoic seawater (*5*). These results robustly confirm the characteristically depleted δ^18^O signature of Precambrian seawater and reinforce the long-term secular trend of increasing seawater δ^18^O values throughout Earth’s history.

The depleted δ^18^O values of Mesoproterozoic seawater unequivocally demonstrate a substantially higher flux of isotopic exchange reactions in low-temperature Earth surface environments relative to high-temperature crustal/mantle environments. Seawater δ^18^O is mainly controlled by two competing processes: (i) high-temperature alteration (mainly hydrothermal alteration at mid-ocean ridges, as well as subducted water recycling), which enriches seawater δ^18^O, and (ii) low-temperature alteration (mainly continental weathering but also submarine weathering), which depletes seawater δ^18^O (*3*, *4*, *12*). The enhanced isotopic exchange flux in surface environments was likely driven by relatively higher temperature, elevated atmospheric oxygen, as well as widespread ultramafic rocks. Our reconstructed Mesoproterozoic seawater temperatures (23.3° to 28.4°C) are substantially lower than Archean estimates [55° to 85°C; (*61*)] but notably higher than most Phanerozoic values (*62*), consistent with elevated *p*CO_2_ conditions (*63*). The relatively warm surface temperature, along with increasing atmospheric oxygen (*64*), and widespread weatherable ultramafic rocks on the continents would have promoted intense continental weathering, as evidenced by the marked δ^18^O difference between Mesoproterozoic marine shales [10 to 15‰; (*11*)] and fresh submarine basalt [5.7 ± 0.2‰; (*65*)]. Concurrently, isotopic exchange in high-temperature crustal/mantle environments may have been suppressed because of reduced spreading rates. The aggregated supercontinent (Columbia-Rodinia) during the Meso- to Neoproterozoic (*66*) resulted in elevated mantle temperatures [1500° to 1600°C versus present-day 1350°C; (*67*)], leading to a weakened lithosphere and a slower spreading rate (*68*). These conditions produced an unusually thick oceanic crust [25 to 35 km versus modern ~7 km; (*66*)]. The relatively slower spreading rate can also be verified by a relatively orogenic quiescence in the Mesoproterozoic, as evidenced by thinner active crust (40 to 45 km) compared to both Phanerozoic and Paleoproterozoic-Early Archean systems (*69*). The diminished spreading rate would have reduced high-temperature alteration at mid-ocean ridges, thereby amplifying the relative influence of continental weathering on seawater δ^18^O values (*2*). In summary, the δ^18^O depletion observed in Mesoproterozoic seawater likely reflects the combined effects of intensified low-temperature continental weathering and attenuated high-temperature submarine alteration. However, precise quantification of their relative contributions requires further constraints on weathering intensities and spreading rates.

## MATERIALS AND METHODS

### Materials

We collected 13 limestones, and two shale samples were from the Xiahuayuan area in Hebei Province, North China (fig. S1). Initially, one shale and eight limestone concretion samples were collected from the middle of unit 1 of Pt_2_*x* at the Xiajiagou outcrop (XJG, 115° 14′ 53.10″ E, 40° 27′ 31.74″ N; fig. S3). In addition, one shale and five stromatolitic limestone samples were collected from the top of unit 1 and the upper part of unit 2 of Pt_2_*x* at the Huangtugang outcrop (HTG, 115° 13′ 21.72″ E, 40° 27′ 10.08″ N; fig. S3), respectively. We estimated the deposition rate of Pt_2_*x* in the Xiahuayuan area to be ~6.25 m/Ma based on the zircon ages of the bentonite and tuff layers in units 2 and 3, respectively (*27*). The vertical distance from the tuff layers in unit 2 to the limestone concretions is ~150 m, suggesting that the geological age of limestone samples is ~1360 Ma. We analyzed 13 limestone samples that were for petrological and geochemical studies, and two shale samples were analyzed for organic petrography and reflectance.

### Petrology and XRD

We analyzed petrology and XRD at the State Key Laboratory of Petroleum Resources and Engineering, China University of Petroleum (Beijing). Petrological observations of limestone samples were performed using a polarizing microscope (Nikon LV100N POL) and a cathodoluminometer (BII CLF-2) operating at a current of 330 μA and a voltage of 10 kV. Calcite grain size was measured on the basis of microscopic images of limestone samples, with the average grain size for each sample calculated from at least 40 measurements of typical calcite grains. XRD analysis was performed using a Philips X’pert PRO MRD System, with operating conditions of 40 mA and 40 kV. Detailed XRD data and calcite grain size are presented in table S1.

### Equivalent vitrinite reflectance

We performed organic petrography analyses using a Leica DM4500P microscope at the State Key Laboratory of Petroleum Resources and Engineering, China University of Petroleum (Beijing) and obtained reflectance values using the Hilgers Technisches Büero Fossil system, with a 1σ SD of less than 0.2%. The equivalent vitrinite reflectance values for vitrinite-like macerals and solid bitumen were calculated using the empirical equations (*70*, *71*)

$$E_{q}VR_{o}=1.07\times \text{VLM}R_{o}-0.18$$ (1)

$$E_{q}VR_{o}=0.87\times \text{SB}R_{o}+0.25$$ (2)

where E_q_V*R*_o_ is equivalent vitrinite reflectance, VLM*R*_o_ is the reflectance of vitrinite-like maceral, and SB*R*_o_ is the reflectance of solid bitumen.

### Carbonate clumped isotope measurement and temperature calibration

#### Definition of carbonate clumped isotope

Clumped isotopes refer to naturally occurring isotopologs that contain more than one heavy isotope (i.e., a rare isotope) (*14*). Carbonate clumped isotopes specifically refer to isotopologs in CO_2_ derived during the phosphoric acid digestion of carbonate minerals that contain two or more heavy isotopes (e.g., ^13^C, ^18^O, and ^17^O). Current research mainly focuses on mass 47 CO_2_ isotopologs (mostly ^13^C^18^O^16^O but also including ^12^C^17^O^18^O and ^13^C^17^O^17^O) and mass 48 CO_2_ isotopologs (mostly ^12^C^18^O^18^O but also including ^13^C^18^O^17^O) isotopologs (*14*, *18*). A clumped isotope value (e.g., Δ_47_ and Δ_48_) is quantified using the Δ*_i_*, which measures the abundance of clumped isotopolog *i*, relative to the abundance predicted for a stochastic (random) distribution (*72*). Δ_47_ and Δ_48_ were calculated using Eqs. 3 and 4, respectively

$$\Delta_{47}=\left[\left(\frac{R_{47}}{R_{47}^{*}}-1\right)-\left(\frac{R_{46}}{R_{46}^{*}}-1\right)-\left(\frac{R_{45}}{R_{45}^{*}}-1\right)\right]\times 1000$$ (3)

$$\Delta_{48}=\left[\left(\frac{R_{48}}{R_{48}^{*}}-1\right)-2\left(\frac{R_{46}}{R_{46}^{*}}-1\right)\right]\times 1000$$ (4)

$$R_{45}^{*}=R_{13}+\left(2\times R_{17}\right)$$ (5)

$$R_{46}^{*}=\left(2\times R_{18}\right)+\left(2\times R_{13}\times R_{17}\right)+{\left(R_{17}\right)}^{2}$$ (6)

$$\begin{array}{c} R_{47}^{*}=\left(2\times R_{13}\times R_{18}\right)+\left(2\times R_{17}\times R_{18}\right)+\left[R_{13}\times {\left(R_{17}\right)}^{2}\right] \end{array}$$ (7)

$$R_{48}^{*}=R_{18}^{2}+2\times R_{13}\times R_{17}\times R_{18}$$ (8)

where *R*_45_, *R*_46_, *R*_47_, and *R*_48_ are measured abundance ratios relative to mass 44, and R^*^_45_, R^*^_46_, R^*^_47_, and R^*^_48_ are the ratios that would be expected for the random or stochastic distribution of isotopes among isotopologs. *R*_13_ and *R*_18_ are the ratios for ^13^C/^12^C and ^18^O/^16^O, respectively.

#### Sample preparation and measurement process

We used an agate mortar and pestle to grind bulk rock samples to a powder with a mesh size smaller than 100 mesh at room temperature. Next, we extracted the powders using dichloromethane for 24 hours to remove any soluble organic matter. Following this, we treated the samples with 3% H_2_O_2_ to eliminate any residual organic matter using the method described in (*73*), and the suspended solids (mainly clays) during the H_2_O_2_ treatment were poured off to remove clay materials.

We then conducted dual-laboratory carbonate clumped isotope analyses using the MAT 253 Plus isotope ratio mass spectrometer (IRMS) at the CIL (Beijing) and the Thermo MAT 253 IRMS at the IGG (Chinese Academy of Sciences), both interfaced with Isotopologue Batch Extraction system (IBEX) for automated sample processing. The IBEX automated ~10 mg of carbonate powder digestion in 105% H_3_PO_4_ at 90°C, followed by a three-stage purification: (i) water removal at −80°C (trap 1), (ii) gas chromatography through a silver wool sulfide trap and −35°C gas chromatography (GC) column [1 m × 2.5 mm inner diameter (ID)] with helium carrier (35 ml/min, 2 psi/13.8 kPa), and (iii) final cryofocusing at −192°C before IRMS introduction. We alternately measured signal intensities at mass/charge ratio (*m/z*) 44 to 49 for both sample and reference gases following established methods (*72*).

#### Data processing and standardization

We processed raw data (δ^45–49^) and standardized clumped isotope measurements following established methods (*74*). The main steps are as follows: (i) At the IGG, we corrected raw Δ_47_ values for nonlinearity using pressure baseline (PBL) correction (*75*), monitoring peak scans at mass 44 beam intensities between 12 and 18 V. We then applied a negative background range of −10 to −30 mV for baseline correction (*76*). At the CIL, we used a MAT 253 Plus mass spectrometer equipped with six Faraday cups (*m/z* 44 to 49) and an additional 47.5 half cup to monitor the background baseline. (ii) We calculated the δ^13^C, δ^18^O, and raw Δ_47_ and Δ_48_ values for each sample following a previously described method (*72*). (iii) We corrected the δ^47^-Δ_47_ and δ^48^-Δ_48_ values nonlinearity with carbonate standards ETH1 to ETH4 provided by ETH Zürich. (iv) We established the transfer function between the raw Δ_47_ values and the I-CDES reference frame using the carbonate reference materials (ETH1 to ETH4) (*42*). (v) The raw Δ_47_ values of the samples were converted to the I-CDES reference frame using the transfer function established above. We used routine running internal standards [NB4 (Δ_47(I-CDES90)_ = 0.389 ± 0.003‰) and P1 (Δ_47(I-CDES90)_ = 0.616 ± 0.005‰)] to monitor the long-term stability of the IRMS.

We processed raw data using Easotope software (*77*) at the CIL, where the PBL correction and linear correction were applied, and the Δ_47(raw)_ and Δ_48(raw)_ values were converted into the I-CDES and CDES reference system, respectively. The corrected clumped isotope values of the repeated samples measured at the two laboratories were quite similar, with a Δ_47_ difference of less than 0.01‰ (table S2).

#### Clumped isotope temperature calibration

The clumped isotope temperatures (T-Δ_47_ and T-Δ_48_) were calibrated using Eqs. 9 and 10, respectively (*41*, *44*)

$$\begin{array}{c} \Delta_{47\left(I-\text{CDES}90\right)}=0.0391\left(\pm 0.0004\right)\times 10^{6}/T^{2}+0.154\left(\pm 0.0004\right)\\ \left(R^{2}=0.97\right) \end{array}$$ (9)

$$\begin{array}{c} \Delta_{48\left(\text{CDES}90\right)}=0.0142\left(\pm 0.0012\right)\times 10^{6}/T^{2}+0.088\left(\pm 0.0014\right)\\ \left(R^{2}=0.96\right) \end{array}$$ (10)

#### Error analysis of standards and samples

All samples, except for the XJG-1, were analyzed between three and nine times (with an average of six times) to ensure reproducibility. We quantified the uncertainties of the Δ_47_ values for both samples and standards using the SE. The SE of the Δ_47_ measurements showed ranges of 0.003 to 0.011‰ for carbonate standards (table S3) and 0.004 to 0.014‰ for limestone samples (Table 1). The uncertainties of clumped isotope temperature were expressed as the average of the positive and negative errors. The original acquisition of the δ^45–49^ data for both samples and standards are presented in data S1 and S2, and detailed data on the δ^13^C, δ^18^O, and clumped isotopes (Δ_47_ and Δ_48_) for limestone samples are presented in table S4.

## Supplementary Materials

### This PDF file includes:

Figs. S1 to S8 Tables S1 to S4 Data S1 and S2 References
