# Supplementary material for: Oxygen isotope composition of Mesoproterozoic (~1360 Ma) seawater constrained by clumped isotopes of North China limestones
Source: Sci Adv. 2025 Oct 17;11(42):eadu6693. doi: 10.1126/sciadv.adu6693 (PMC12533587; doi:10.1126/sciadv.adu6693)
Supplement: Supplementary file 1 — Figs. S1 to S8 Tables S1 to S4 Data S1 and S2 References [file sciadv.adu6693_sm.pdf]

Science Advances
AAAS

Supplementary Materials for

Oxygen isotope composition of Mesoproterozoic (~1360 Ma) seawater constrained by clumped isotopes of North China limestones

Pingping Li *et al.*

Corresponding author: Pingping Li, lpp@cup.edu.cn; Fang Hao, haofang@upc.edu.cn

Sci. Adv. **11**, eadu6693 (2025)
DOI: 10.1126/sciadv.adu6693

This PDF file includes:

Figs. S1 to S8
Tables S1 to S4
Data S1 and S2
References

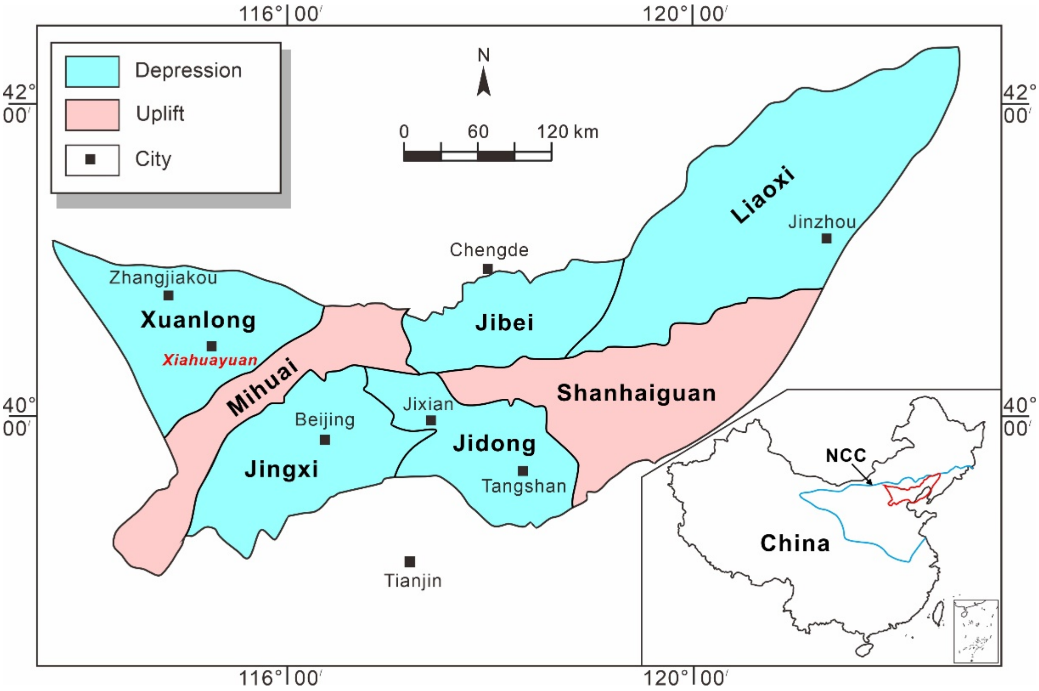

Fig. S1. Sampling location of Xiahuayuan in the Xuanlong depression. Tectonic units of the Yanliao faulted depressions within the North China Craton (NCC) was modified from ref. (*79*).

Reprinted from Petroleum Science Bulletin, Vol. 1, Tieguan Wang, Ningning Zhong, Chunjiang Wang, Yixiu Zhu, Yan Liu, Daofu Song, Source beds and oil entrapment-alteration histories of fossil-oil-reservoirs in the Xiamaling Formation Basal Sandstone, Jibei Depression, 24-37, Copyright (2016), with permission from China University of Petroleum, Beijing.

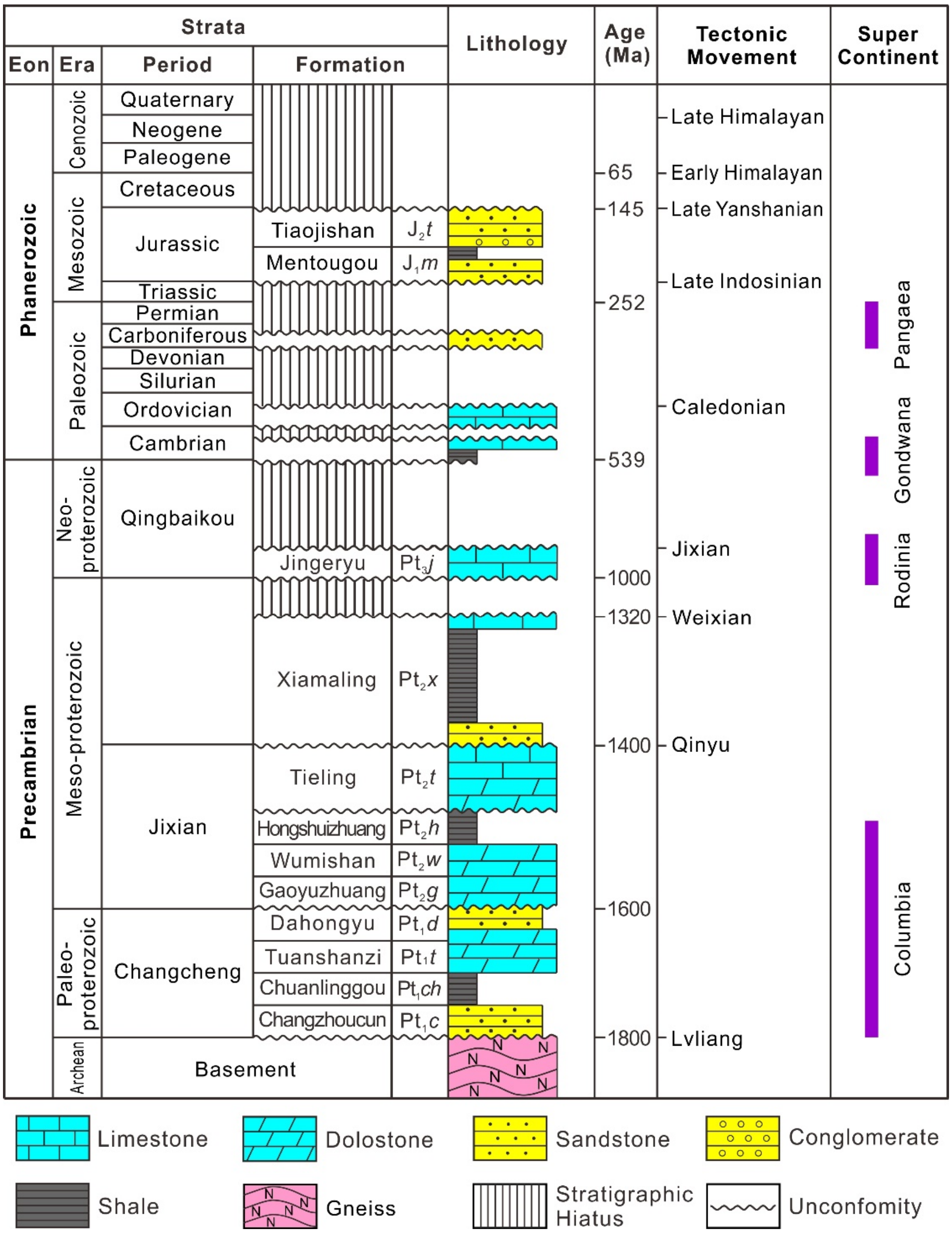

Fig. S2. Generalized stratigraphy and main tectonic events in North China Craton. The supercontinent assembly history follows ref. (*80*). The Jurassic units unconformably overlie the Mesoproterozoic Xiamaling Formation in the study area.

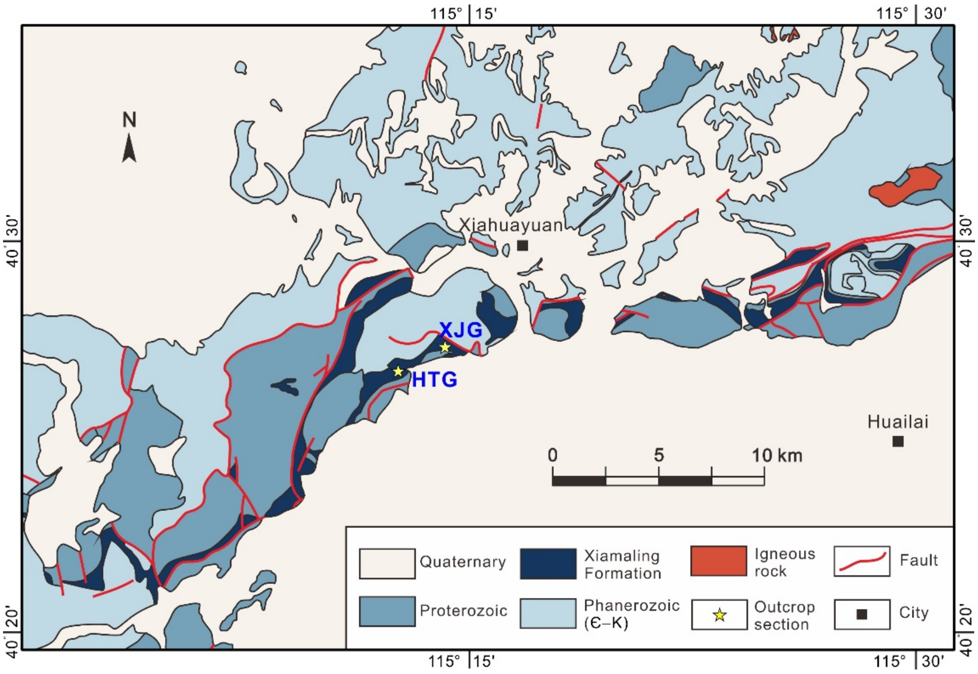

Fig. S3. Locations of the Xiajiagou (XJG) and Huangtugang (HTG) outcrops. The simplified geological map of the Xiahuayuan area was modified from ref. (*25*).

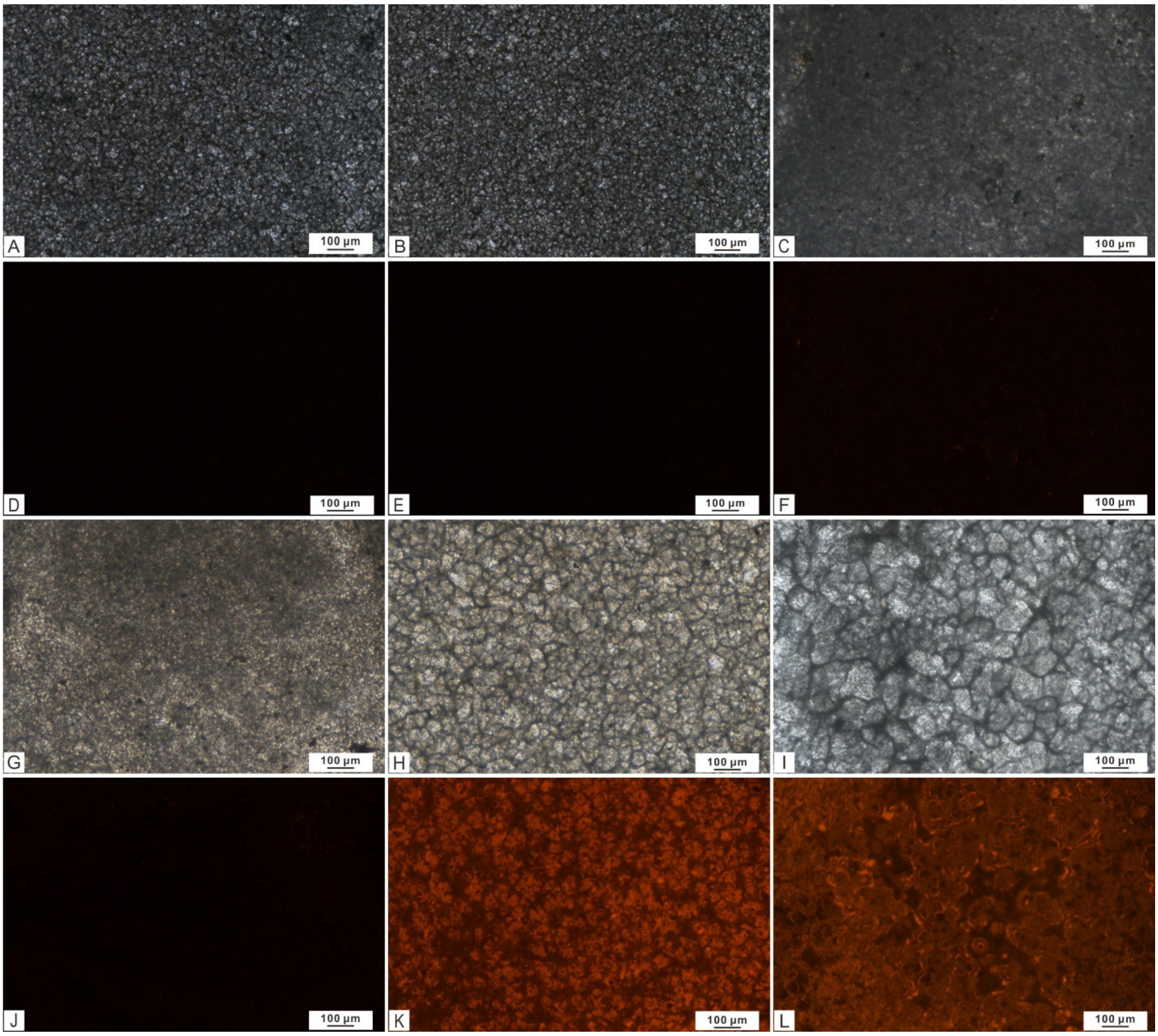

Fig. S4. Typical photomicrographs of limestones. **(A, D)** Sample XJG-3, limestone concretion exhibiting non-luminescence. **(B, E)** Sample XJG-7, limestone concretion exhibiting non-luminescence. **(C, F)** Sample HTG-9, stromatolitic limestone exhibiting non-luminescence. **(G, J)** Sample HGT-10, stromatolitic limestone exhibiting non-luminescence. **(H, K)** Sample XJG-1, limestone concretion exhibiting bright yellow cathodoluminescence. **(I, L)** Sample XJG-10, limestone concretion exhibiting bright yellow cathodoluminescence.

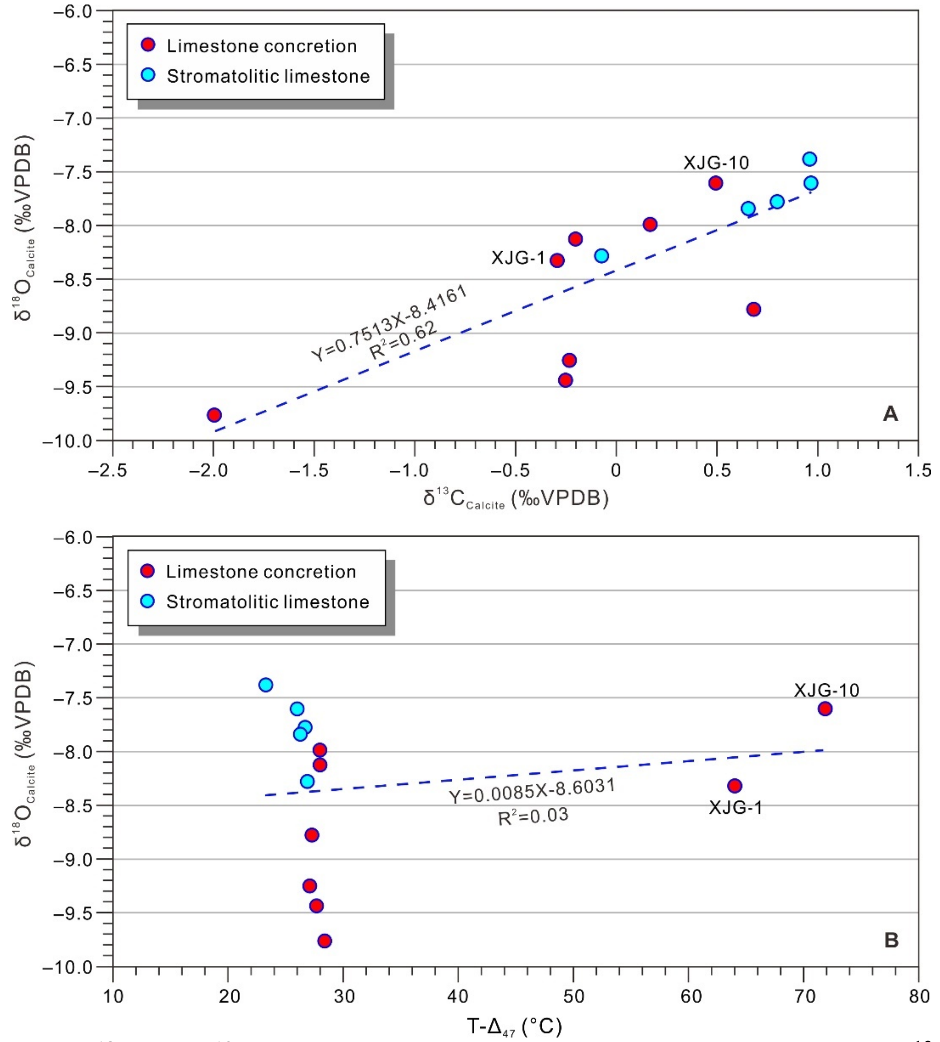

Fig. S5. Plot of $\delta^{18}O$ vs. $\delta^{13}C$ and clumped isotope temperature (T-Δ_{47}). (**A**) $\delta^{13}C$ and $\delta^{18}O$ values exhibit an approximate positive correlation. (**B**) T-Δ47 values show no linear relationship with $\delta^{18}O$ values of limestone samples. Excluding two high-temperature samples (XJG-1 and XJG-10), the remaining samples demonstrate remarkable consistency and stability in their T-Δ_{47} values.

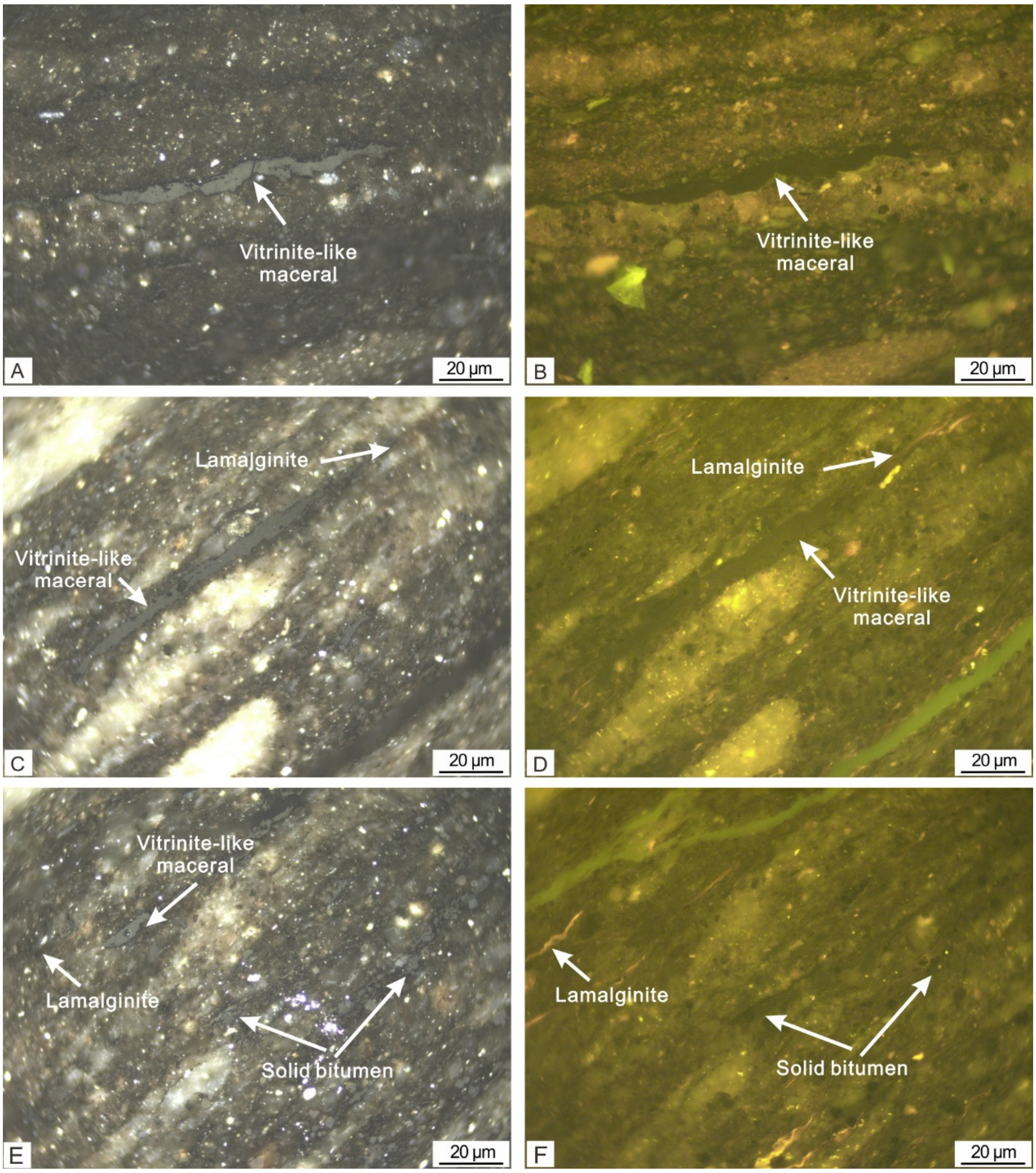

Fig. S6. Photomicrographs of organic matters in shales. A, C and E are under reflected light, while B, D and F are under fluorescence light. (**A**, **B**) Sample XJG-1, showing vitrinite-like maceral. (**C**, **D**) Sample HTG-1, displaying vitrinite-like maceral and lamalginite. (**E**, **F**) Sample HTG-1, displaying vitrinite-like maceral, lamalginite, and solid bitumen.

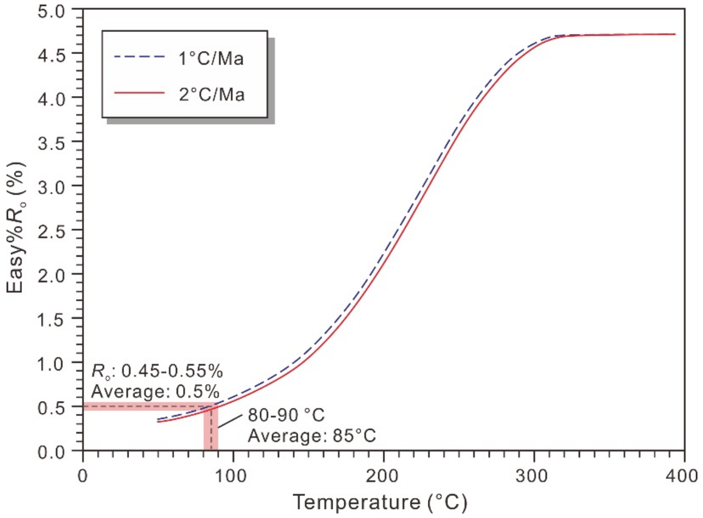

Fig. S7. Vitrinite reflectance (*R*o) vs. temperature. *R*o values were calculated according to the Easy*R*o model (*55*), and average *R*o value for the two shale samples is 0.50%, corresponding to an average maximum burial temperature of 85°C.

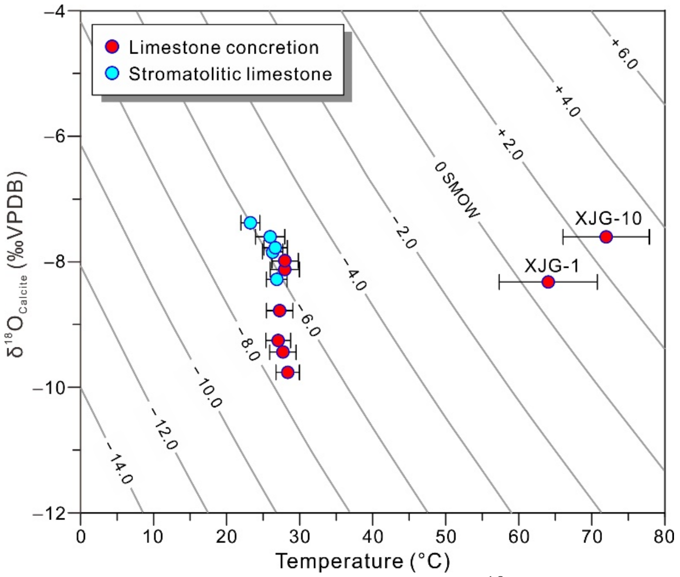

Fig. S8. Oxygen isotopes of Mesoproterozoic seawater. $\delta^{18}O$ values were calculated using the equation previously described in ref. (*15*): $10^3 \ln\alpha_{calcite\text{-}water} = 18.03 \times 10^3/T - 32.42$.

Sample ID	Strata	Type	Grain size of calcite (μm)	Mineral component (%)				
				Calcite	Dolomite	Quartz	Feldspar	Clay
XJG-1	Pt_2x	LC	73.4±7.4	89.3	0	10.7	0	0
XJG-2	Pt_2x	LC	34.1±3.8	86.1	0	11.3	0	2.6
XJG-3	Pt_2x	LC	23.1±2.9	81.4	0	18.6	0	0
XJG-4	Pt_2x	LC	29.2±2.2	87.3	0	12.7	0	0
XJG-6	Pt_2x	LC	23.2±2.2	79.6	4.3	16.1	0	0
XJG-7	Pt_2x	LC	26.6±1.8	84.9	0	15.1	0	0
XJG-8	Pt_2x	LC	31.4±2.5	87.3	0	12.7	0	0
XJG-10	Pt_2x	LC	108.6±7.0	83	5.1	11.9	0	0
HTG-1	Pt_2x	SL	6.5±1.2	94.8	0	5.2	0	0
HTG-7	Pt_2x	SL	9.8±0.8	92.9	0	7.1	0	0
HTG-9	Pt_2x	SL	14.5±1.9	93.1	0	6.9	0	0
HTG-10	Pt_2x	SL	15.0±1.3	93.9	0	6.1	0	0
HTG-14	Pt_2x	SL	7.4±1.3	88.3	0	5.6	0	6.1

Table S1. Mineral compositions and grain sizes in limestones. The mineral compositions were obtained from XRD analyses. LC represents limestone concretion, and SL represents stromatolitic limestone.

Sample	$\delta^{13}C_{VPDB}$ (‰)			$\delta^{18}O_{VPDB}$ (‰)			$\Delta_{47\ (I\text{-}CDES90)}$ (‰)		
	CIL	IGG	Difference value	CIL	IGG	Difference value	CIL	IGG	Difference value
XJG-2	0.164(3)	0.172(3)	0.008	–7.984(3)	–7.990(3)	0.006	0.584(3)	0.585(3)	0.001
XJG-3	0.671(2)	0.688(3)	0.017	–8.782(2)	–8.777(3)	0.005	0.585(2)	0.588(3)	0.003
XJG-4	–0.193(2)	–0.209(3)	0.016	–8.119(2)	–8.129(3)	0.010	0.592(2)	0.580(3)	0.012
XJG-8	–0.254(3)	–0.252(3)	0.002	–9.442(3)	–9.433(3)	0.009	0.587(3)	0.586(3)	0.001
HTG-7	–0.076(6)	–0.073(3)	0.003	–8.279(6)	–8.285(3)	0.006	0.587(6)	0.591(3)	0.004
HTG-9	0.650(3)	0.659(3)	0.009	–7.838(3)	–7.845(3)	0.007	0.588(3)	0.591(3)	0.003

Table S2. Comparison of $\delta^{13}C$, $\delta^{18}O$ and Δ_{47} values measured in two laboratories. CIL represents Clumped Isotopes Laboratory, and IGG represents Institute of Geology and Geophysics. The numbers in parentheses represent sample replicates.

Standard	CIL (MAT 253 plus)			IGG (MAT 253)		
	n	$\Delta_{47\ (I\text{-}CDES90)}$ (‰)	$\Delta_{47(raw)}$SE	n	$\Delta_{47\ (I\text{-}CDES90)}$ (‰)	$\Delta_{47(raw)}$SE
ETH1	4	0.207	0.009	8	0.205	0.007
ETH2	11	0.208	0.005	8	0.208	0.005
ETH3	18	0.615	0.003	6	0.614	0.006
ETH4	3	0.456	0.010	2	0.452	0.011
IAEA-C2	12	0.644	0.004	/	/	/
NB4	6	0.383	0.005	10	0.386	0.004
P1	/	/	/	20	0.618	0.003

Table S3. Comparison of Δ_{47} values of carbonate standards measured in two laboratories. Column "n" is the number of Δ_{47} analyses for each standard, and SE is standard error.

Sample ID	Date	Round	$\delta^{13}C$ (‰VPDB)	$\delta^{18}O$ (‰VPDB)	Δ_{47} (I-CDES90, ‰)	Δ_{48} (I-CDES90, ‰)
XJG–1	2023/11/29	R1 (CIL)	–0.304±0.008	–8.331±0.009	0.510±0.011	0.188±0.062
	2023/11/30	R2 (CIL)	–0.283±0.007	–8.315±0.008	0.482±0.010	0.221±0.055
XJG–2	2023/11/24	R1 (CIL)	0.164±0.007	–7.982±0.009	0.588±0.009	0.228±0.054
	2023/11/25	R2 (CIL)	0.151±0.009	–7.976±0.008	0.594±0.011	0.253±0.047
	2023/11/29	R3 (CIL)	0.177±0.008	–7.994±0.010	0.570±0.010	/
	2025/01/21	R4 (IGG)	0.175±0.007	–7.979±0.012	0.575±0.013	/
	2025/01/22	R5 (IGG)	0.180±0.010	–7.991±0.011	0.588±0.012	/
	2025/01/23	R6 (IGG)	0.162±0.009	–8.001±0.012	0.590±0.012	/
XJG–3	2023/11/16	R1 (CIL)	0.668±0.008	–8.778±0.010	0.577±0.007	/
	2023/11/17	R2 (CIL)	0.673±0.009	–8.785±0.008	0.593±0.009	
	2024/01/27	R3 (IGG)	0.683±0.007	–8.768±0.011	0.573±0.010	
	2024/01/28	R4 (IGG)	0.696±0.009	–8.783±0.011	0.601±0.009	
	2024/01/29	R5 (IGG)	0.685±0.008	–8.780±0.012	0.591±0.010	
XJG–4	2023/11/24	R1 (CIL)	–0.197±0.009	–8.127±0.008	0.595±0.009	/
	2023/11/25	R2 (CIL)	–0.189±0.009	–8.111±0.009	0.588±0.009	
	2024/01/27	R3 (IGG)	–0.201±0.010	–8.128±0.011	0.597±0.014	
	2024/01/28	R4 (IGG)	–0.219±0.007	–8.125±0.010	0.568±0.011	
	2024/01/29	R5 (IGG)	–0.207±0.008	–8.134±0.011	0.575±0.011	
XJG–6	2023/11/16	R1 (CIL)	–1.991±0.008	–9.772±0.012	0.565±0.014	0.213±0.067
	2023/11/17	R2 (CIL)	–1.971±0.007	–9.768±0.011	0.577±0.014	/
	2023/11/29	R3 (CIL)	–1.993±0.008	–9.751±0.011	0.588±0.012	0.252±0.042
	2024/08/07	R4* (CIL)	–2.021±0.005	–9.759±0.009	0.590±0.009	0.245±0.027
	2024/08/07	R5* (CIL)	–1.997±0.009	–9.748±0.010	0.597±0.010	0.265±0.024
	2024/08/13	R6* (CIL)	–2.011±0.008	–9.767±0.008	0.588±0.009	0.222±0.035
XJG–7	2023/11/16	R1 (CIL)	–0.258±0.007	–9.245±0.011	0.598±0.010	0.209±0.045
	2023/11/17	R2 (CIL)	–0.223±0.007	–9.260±0.009	0.593±0.011	0.255±0.055
	2023/11/29	R3 (CIL)	–0.235±0.009	–9.241±0.010	0.572±0.010	/
	2024/08/07	R4* (CIL)	–0.227±0.006	–9.247±0.008	0.583±0.010	0.265±0.041
	2024/08/07	R5* (CIL)	–0.231±0.005	–9.265±0.008	0.590±0.009	0.234±0.017
	2024/08/13	R6* (CIL)	–0.229±0.006	–9.258±0.010	0.595±0.010	0.249±0.026
XJG–8	2023/11/16	R1 (CIL)	–0.267±0.006	–9.438±0.007	0.596±0.011	/
	2023/11/17	R2 (CIL)	–0.250±0.007	–9.453±0.008	0.574±0.011	
	2023/11/29	R3 (CIL)	–0.246±0.007	–9.435±0.008	0.590±0.010	
	2025/01/21	R4 (IGG)	–0.260±0.008	–9.431±0.011	0.574±0.011	
	2025/01/22	R5 (IGG)	–0.256±0.008	–9.445±0.009	0.593±0.012	
	2025/01/23	R6 (IGG)	–0.241±0.010	–9.424±0.013	0.589±0.011	
XJG–10	2023/11/24	R1 (CIL)	0.496±0.007	–7.608±0.010	0.502±0.009	/
	2023/11/25	R2 (CIL)	0.479±0.007	–7.611±0.009	0.483±0.011	0.175±0.069
	2023/11/29	R3 (CIL)	0.505±0.008	–7.597±0.009	0.462±0.010	0.216±0.073
HTG–1	2023/11/16	R1 (CIL)	0.972±0.007	–7.604±0.010	0.599±0.013	/
	2023/11/17	R2 (CIL)	0.952±0.006	–7.588±0.010	0.595±0.011	0.214±0.113
	2023/11/30	R3 (CIL)	0.977±0.007	–7.612±0.011	0.580±0.012	0.256±0.107
	2023/11/30	R4 (CIL)	0.962±0.006	–7.609±0.009	0.590±0.010	0.236±0.073

HTG–7	2023/11/24	R1 (CIL)	–0.081±0.008	–8.286±0.009	0.590±0.012	0.236±0.058
	2023/11/25	R2 (CIL)	–0.092±0.009	–8.268±0.010	0.572±0.011	0.269±0.063
	2023/11/30	R3 (CIL)	–0.089±0.008	–8.278±0.011	0.596±0.011	/
	2024/01/27	R4 (IGG)	–0.064±0.007	–8.285±0.012	0.601±0.012	/
	2024/01/28	R5 (IGG)	–0.068±0.010	–8.282±0.010	0.573±0.010	/
	2024/01/29	R6 (IGG)	–0.059±0.009	–8.276±0.010	0.591±0.010	/
	2024/08/07	R7* (CIL)	–0.072±0.005	–8.293±0.008	0.592±0.009	0.265±0.017
	2024/08/07	R8* (CIL)	–0.081±0.005	–8.291±0.009	0.587±0.010	0.234±0.031
	2024/08/13	R9* (CIL)	–0.067±0.006	–8.270±0.008	0.593±0.009	0.239±0.022
HTG–9	2023/11/24	R1 (CIL)	0.664±0.007	–7.845±0.010	0.579±0.012	/
	2023/11/25	R2 (CIL)	0.641±0.006	–7.831±0.011	0.596±0.010	
	2023/11/30	R3 (CIL)	0.644±0.006	–7.837±0.009	0.589±0.010	
	2025/01/21	R4 (IGG)	0.663±0.009	–7.834±0.012	0.595±0.012	
	2025/01/22	R5 (IGG)	0.645±0.008	–7.849±0.010	0.586±0.010	
	2025/01/23	R6 (IGG)	0.670±0.009	–7.853±0.013	0.593±0.014	
HTG–10	2023/11/16	R1 (CIL)	0.979±0.006	–7.395±0.010	0.592±0.011	0.225±0.104
	2023/11/17	R2 (CIL)	0.958±0.007	–7.378±0.012	0.605±0.011	0.259±0.071
	2023/11/24	R3* (CIL)	0.966±0.007	–7.391±0.008	0.601±0.010	0.264±0.094
	2024/08/13	R4* (CIL)	0.959±0.005	–7.385±0.007	0.611±0.009	0.249±0.051
	2024/08/13	R5* (CIL)	0.942±0.006	–7.370±0.009	0.597±0.010	0.211±0.067
	2024/08/13	R6* (CIL)	0.951±0.007	–7.373±0.008	0.588±0.008	0.257±0.039
HTG–14	2023/11/24	R1 (CIL)	0.788±0.009	–7.773±0.011	0.597±0.011	/
	2023/11/25	R2 (CIL)	0.805±0.008	–7.788±0.011	0.581±0.012	
	2023/11/30	R3 (CIL)	0.801±0.006	–7.772±0.009	0.590±0.011	

Table S4. $\delta^{13}C$, $\delta^{18}O$, Δ_{47}, and Δ_{48} values per measurement. * denote the Δ_{48} were measured using the method previously described in ref. (*18*).

Data S1. Raw clumped isotope data of samples and standards measured at the CIL.

Date	Type	ID	δ^{45} (raw)	δ^{46} (raw)	δ^{47} (raw)	δ^{48} (raw)	δ^{49} (raw)	Δ_{47} (raw)	Δ_{47} (raw)SD	Δ_{48} (raw)	Δ_{48} (raw)SD
2023/11/16	sample	HTG-1	22.764	22.928	44.873	42.933	37.538	–0.209	0.013	–0.876	0.189
2023/11/16	sample	HTG-10	24.213	24.103	49.077	48.542	46.380	–0.191	0.011	–1.029	0.104
2023/11/16	sample	XJG-3	23.148	23.784	45.445	40.129	31.054	–0.197	0.007	–0.975	0.264
2023/11/16	sample	XJG-6	21.732	22.126	43.648	41.764	36.409	–0.214	0.014	–1.169	0.067
2023/11/16	sample	XJG-7	22.888	22.402	45.926	44.834	33.118	–0.214	0.010	–1.180	0.045
2023/11/16	sample	XJG-8	23.751	23.984	44.710	39.150	32.592	–0.215	0.011	–1.261	0.243
2023/11/17	sample	HTG-1	22.741	22.914	44.862	42.022	35.065	–0.202	0.011	–1.170	0.073
2023/11/17	sample	HTG-10	24.203	24.112	49.105	48.264	41.354	–0.196	0.011	–1.185	0.071
2023/11/17	sample	XJG-3	23.162	23.851	45.430	41.236	36.544	–0.218	0.009	–0.830	0.324
2023/11/17	sample	XJG-6	21.722	22.253	43.644	41.522	32.582	–0.229	0.014	–1.203	0.027
2023/11/17	sample	XJG-7	22.957	22.310	45.941	44.640	43.789	–0.196	0.011	–1.198	0.055
2023/11/17	sample	XJG-8	23.744	23.945	44.690	40.021	33.453	–0.196	0.011	–1.407	0.197
2023/11/24	sample	HTG-14	22.809	23.134	41.895	38.770	28.562	–0.218	0.011	–1.026	0.397
2023/11/24	sample	HTG-7	23.003	22.965	46.626	46.743	30.691	–0.223	0.012	–1.187	0.058
2023/11/24	sample	HTG-9	23.101	23.909	44.182	41.835	31.264	–0.216	0.012	–1.165	0.331
2023/11/24	sample	XJG-10	23.741	23.969	48.413	48.801	38.800	–0.249	0.009	–0.735	0.069
2023/11/24	sample	XJG-2	21.072	22.133	41.891	40.971	32.584	–0.194	0.009	–1.121	0.054
2023/11/24	sample	XJG-4	23.375	23.105	42.867	41.366	30.565	–0.216	0.009	–1.412	0.216
2023/11/25	sample	HTG-14	22.814	23.140	41.885	38.751	36.979	–0.226	0.012	–1.354	0.235
2023/11/25	sample	HTG-7	23.082	22.923	46.417	46.256	35.654	–0.208	0.011	–1.138	0.063
2023/11/25	sample	HTG-9	23.095	23.901	44.188	41.825	34.686	–0.195	0.010	–0.921	0.416
2023/11/25	sample	XJG-10	23.736	23.965	48.399	48.100	41.235	–0.232	0.011	–0.912	0.073
2023/11/25	sample	XJG-2	21.085	22.138	41.881	40.669	36.538	–0.220	0.011	–1.179	0.047
2023/11/25	sample	XJG-4	22.372	23.106	42.859	41.350	37.565	–0.201	0.009	–1.303	0.199
2023/11/29	sample	XJG-1	22.893	21.589	45.106	44.226	31.735	–0.239	0.011	–0.803	0.062
2023/11/29	sample	XJG-10	23.747	23.980	45.424	48.770	31.931	–0.220	0.010	–1.139	0.217
2023/11/29	sample	XJG-2	21.074	22.123	41.879	40.920	36.540	–0.201	0.010	–1.007	0.191
2023/11/29	sample	XJG-6	21.705	22.086	43.446	42.845	37.862	–0.196	0.014	–1.276	0.207
2023/11/29	sample	XJG-7	22.938	22.218	45.933	44.314	37.455	–0.187	0.013	–0.994	0.228
2023/11/29	sample	XJG-8	23.742	23.963	44.697	39.072	39.558	–0.209	0.010	–1.326	0.186
2023/11/30	sample	HTG-1	22.781	22.901	44.860	41.634	33.594	–0.187	0.012	–1.186	0.107
2023/11/30	sample	HTG-1	22.776	22.913	44.849	42.151	40.154	–0.213	0.010	–1.172	0.073
2023/11/30	sample	HTG-14	22.817	21.134	41.880	38.815	40.560	–0.202	0.011	–1.168	0.307
2023/11/30	sample	HTG-7	23.042	22.949	46.662	46.587	27.593	–0.194	0.011	–1.061	0.294
2023/11/30	sample	HTG-9	23.102	23.915	44.175	41.364	39.482	–0.221	0.010	–1.287	0.162
2023/11/30	sample	XJG-1	22.885	21.585	45.094	44.104	39.652	–0.216	0.010	–0.982	0.055
2023/11/10	standard	ETH1	24.235	27.993	46.732	64.325	70.381	–0.292	0.009	–0.502	0.124
2023/11/10	standard	ETH1	24.231	28.062	46.771	69.510	83.771	–0.318	0.010	–0.546	0.158
2023/11/10	standard	ETH2	12.821	9.852	22.630	19.322	7.524	–0.371	0.013	–0.660	0.122
2023/11/10	standard	ETH2	12.830	9.857	22.642	19.100	4.343	–0.352	0.009	–0.681	0.097
2023/11/10	standard	ETH3	24.782	27.591	53.105	53.470	31.544	–0.177	0.010	–1.405	0.105
2023/11/10	standard	ETH3	24.779	27.580	53.092	56.549	37.431	–0.199	0.011	–1.298	0.112
2023/11/10	standard	ETH3	24.775	27.576	53.107	54.013	28.597	–0.172	0.012	–1.357	0.087
2023/11/10	standard	ETH4	12.153	10.956	22.176	17.487	12.680	–0.296	0.011	–0.923	0.157
2023/11/10	standard	ETH4	12.159	10.940	22.174	20.450	32.428	–0.265	0.008	–0.874	0.114
2023/11/14	standard	ETH2	12.827	9.855	22.639	19.107	4.690	–0.358	0.012	–0.693	0.131

2023/11/14	standard	ETH2	12.831	9.860	22.640	17.459	3.375	–0.343	0.011	–0.614	0.072
2023/11/14	standard	ETH2	12.840	9.877	22.661	19.560	6.728	–0.366	0.010	–0.866	0.114
2023/11/14	standard	ETH3	24.766	27.591	53.107	54.688	41.125	–0.196	0.009	–1.279	0.087
2023/11/14	standard	ETH3	24.789	27.586	53.098	54.073	38.460	–0.180	0.010	–1.291	0.103
2023/11/14	standard	ETH3	24.773	27.601	53.107	53.964	36.486	–0.195	0.010	–1.327	0.065
2023/11/14	standard	ETH3	24.760	27.596	53.090	51.656	33.557	–0.187	0.010	–1.345	0.047
2023/11/14	standard	ETH3	24.781	27.620	53.119	55.325	40.009	–0.198	0.012	–1.288	0.091
2023/11/14	standard	ETH3	24.773	27.605	53.104	51.115	30.636	–0.173	0.012	–1.304	0.065
2023/11/16	standard	IAEA-C2	15.291	20.325	35.883	41.388	13.186	–0.171	0.010	–1.080	0.089
2023/11/16	standard	NB4	21.775	21.088	43.085	42.601	21.879	–0.240	0.012	–0.095	0.056
2023/11/16	standard	IAEA-C2	15.285	20.320	35.876	40.320	10.684	–0.192	0.012	–1.128	0.120
2023/11/17	standard	IAEA-C2	15.276	20.318	35.862	39.208	8.633	–0.173	0.010	–1.151	0.135
2023/11/17	standard	NB4	21.784	21.095	43.103	46.954	9.602	–0.253	0.011	–0.173	0.158
2023/11/17	standard	IAEA-C2	15.282	20.323	35.881	42.208	13.594	–0.159	0.010	–0.930	0.104
2023/11/24	standard	IAEA-C2	15.275	20.316	35.870	33.846	10.680	–0.196	0.011	–1.107	0.113
2023/11/24	standard	NB4	21.793	21.091	43.085	36.233	13.620	–0.271	0.012	–0.153	0.120
2023/11/24	standard	IAEA-C2	15.280	20.332	35.862	31.568	10.325	–0.164	0.010	–1.163	0.113
2023/11/25	standard	IAEA-C2	15.271	20.319	35.856	37.540	8.946	–0.181	0.011	–1.131	0.084
2023/11/25	standard	NB4	21.774	21.080	43.096	51.364	18.117	–0.239	0.012	–0.182	0.092
2023/11/25	standard	IAEA-C2	15.292	20.328	35.865	32.965	6.531	–0.179	0.013	–1.011	0.112
2023/11/29	standard	IAEA-C2	15.285	20.321	35.853	35.794	16.614	–0.154	0.010	–1.090	0.151
2023/11/29	standard	NB4	21.791	21.075	43.110	20.334	16.801	–0.258	0.015	–0.091	0.130
2023/11/29	standard	IAEA-C2	15.288	20.321	35.878	28.471	10.365	–0.188	0.012	–1.123	0.082
2023/11/30	standard	IAEA-C2	15.270	20.330	35.855	36.570	11.642	–0.160	0.010	–1.162	0.157
2023/11/30	standard	NB4	21.780	21.062	43.102	31.282	22.634	–0.242	0.008	–0.073	0.052
2023/11/30	standard	IAEA-C2	15.297	20.334	35.867	36.142	10.380	–0.179	0.009	–1.122	0.147
2024/8/7	sample	HTG-7	22.982	22.959	46.251	46.730	32.558	–0.219	0.009	–1.203	0.017
2024/8/7	sample	HTG-7	22.989	22.956	46.239	46.721	36.544	–0.196	0.010	–1.224	0.031
2024/8/7	sample	XJG-6	21.691	22.055	43.577	41.512	37.655	–0.209	0.009	–1.198	0.027
2024/8/7	sample	XJG-6	21.688	22.050	43.570	41.503	34.508	–0.203	0.010	–1.220	0.024
2024/8/7	sample	XJG-7	22.912	22.411	45.930	44.824	31.264	–0.192	0.010	–1.211	0.041
2024/8/7	sample	XJG-7	22.915	22.402	45.920	44.829	33.456	–0.209	0.009	–1.187	0.017
2024/8/13	sample	HTG-10	24.231	24.111	49.064	48.420	46.212	–0.177	0.010	–1.228	0.094
2024/8/13	sample	HTG-10	24.226	24.106	49.069	48.411	45.308	–0.183	0.009	–1.240	0.051
2024/8/13	sample	HTG-10	24.221	24.114	49.067	48.435	41.357	–0.208	0.010	–1.212	0.067
2024/8/13	sample	HTG-7	22.996	22.957	46.245	46.717	31.566	–0.214	0.008	–1.223	0.022
2024/8/13	sample	XJG-6	21.695	22.044	43.580	41.529	39.102	–0.212	0.009	–1.306	0.035
2024/8/13	sample	XJG-7	22.901	22.415	45.924	44.831	32.159	–0.206	0.010	–1.215	0.026
2024/8/16	sample	HTG-10	24.232	24.108	49.080	47.124	43.684	–0.199	0.008	–1.253	0.039
2024/8/2	standard	ETH1	24.227	28.034	46.717	62.107	66.308	–0.297	0.011	–0.463	0.107
2024/8/2	standard	ETH1	24.234	28.016	46.735	65.411	78.589	–0.331	0.013	–0.585	0.139
2024/8/2	standard	ETH2	12.807	9.849	22.611	18.960	14.761	–0.351	0.008	–0.649	0.148
2024/8/2	standard	ETH2	12.815	9.851	22.628	19.127	3.007	–0.378	0.011	–0.679	0.156
2024/8/2	standard	ETH2	12.820	9.855	22.619	19.150	9.108	–0.360	0.013	–0.691	0.130
2024/8/2	standard	ETH3	24.776	27.594	53.070	53.611	23.097	–0.170	0.012	–1.370	0.177
2024/8/2	standard	ETH3	24.789	27.577	53.046	51.509	40.038	–0.198	0.011	–1.301	0.102
2024/8/2	standard	ETH3	24.764	27.583	53.062	59.032	27.367	–0.178	0.008	–1.353	0.136
2024/8/2	standard	ETH4	12.145	10.933	22.151	17.005	21.209	–0.270	0.009	–0.806	0.170
2024/8/7	standard	ETH2	12.842	9.874	22.646	18.754	2.190	–0.355	0.010	–0.639	0.028

2024/8/7	standard	ETH3	24.771	27.598	53.112	54.176	36.105	–0.202	0.008	–1.230	0.025
2024/8/7	standard	ETH3	24.785	27.581	53.103	54.192	42.472	–0.195	0.010	–1.123	0.023
2024/8/13	standard	ETH2	12.599	9.586	22.861	17.935	1.173	–0.395	0.013	–0.683	0.031
2024/8/13	standard	ETH3	24.755	27.548	53.107	54.180	32.565	–0.188	0.009	–1.247	0.041
2024/8/13	standard	ETH3	24.758	27.557	53.125	54.190	36.581	–0.205	0.007	–1.259	0.035
2024/8/16	standard	ETH2	12.114	9.320	22.803	17.460	7.898	–0.376	0.009	–0.736	0.025
2024/8/16	standard	ETH3	24.760	27.560	53.127	54.169	31.065	–0.213	0.009	–1.270	0.023
2024/8/16	standard	ETH3	24.779	27.571	53.119	54.197	37.580	–0.206	0.010	–1.307	0.030

Data S2. Raw clumped isotope data of samples and standards measured at the IGG.

Date	Type	ID	δ^{45}(raw)	δ^{46}(raw)	δ^{47}(raw)	δ^{48}(raw)	δ^{49}(raw)	Δ_{47}(raw)	Δ_{47}(raw) SD
2024/1/27	sample	HTG-7	20.861	20.104	44.968	46.625	30.613	–0.219	0.012
2024/1/27	sample	XJG-3	21.756	22.132	43.231	37.891	30.267	–0.190	0.010
2024/1/27	sample	XJG-4	20.168	21.051	41.124	42.351	30.561	–0.209	0.014
2024/1/28	sample	HTG-7	20.854	20.007	44.924	46.210	39.483	–0.226	0.010
2024/1/28	sample	XJG-3	21.751	22.124	43.216	37.014	35.691	–0.212	0.009
2024/1/28	sample	XJG-4	20.175	21.034	41.112	42.910	35.697	–0.231	0.011
2024/1/29	sample	HTG-7	21.850	22.995	46.957	46.529	37.581	–0.203	0.010
2024/1/29	sample	XJG-3	21.745	22.129	43.254	41.021	39.652	–0.196	0.010
2024/1/29	sample	XJG-4	20.181	21.038	41.108	42.614	39.560	–0.201	0.011
2024/1/20	standard	ETH1	20.155	25.089	45.336	79.007	75.451	–0.541	0.011
2024/1/20	standard	ETH1	20.147	25.072	45.320	73.685	59.360	–0.550	0.012
2024/1/20	standard	ETH2	8.602	8.593	15.862	12.056	10.034	–0.610	0.012
2024/1/20	standard	ETH2	8.591	8.588	15.845	19.230	15.961	–0.616	0.008
2024/1/20	standard	ETH3	19.783	25.580	45.927	50.620	70.312	–0.170	0.010
2024/1/20	standard	ETH4	8.245	8.523	16.551	13.086	19.203	–0.396	0.012
2024/1/20	standard	NB4	16.660	18.658	35.430	32.591	37.203	–0.355	0.012
2024/1/20	standard	P1	15.463	21.281	37.009	40.201	26.221	–0.171	0.012
2024/1/20	standard	P1	15.445	21.266	37.011	53.007	31.265	–0.188	0.010
2024/1/24	standard	ETH1	20.165	25.083	45.322	84.613	79.381	–0.542	0.010
2024/1/24	standard	ETH1	20.141	25.070	45.306	72.568	73.586	–0.592	0.013
2024/1/24	standard	ETH2	8.593	8.587	15.867	15.935	6.265	–0.592	0.012
2024/1/24	standard	ETH2	8.588	8.573	15.849	16.621	9.834	–0.602	0.012
2024/1/24	standard	ETH3	19.769	25.573	45.932	52.827	73.581	–0.182	0.010
2024/1/24	standard	ETH3	19.758	25.557	45.915	52.198	68.581	–0.210	0.011
2024/1/24	standard	NB4	16.652	18.650	35.445	38.451	42.428	–0.363	0.011
2024/1/24	standard	P1	15.457	21.273	37.010	43.979	44.676	–0.181	0.010
2024/1/24	standard	P1	15.449	21.261	37.027	36.041	20.520	–0.201	0.010
2024/1/25	standard	ETH1	20.158	25.076	45.307	81.223	75.694	–0.539	0.010
2024/1/25	standard	ETH1	20.146	25.079	45.317	75.631	79.935	–0.551	0.012
2024/1/25	standard	ETH2	8.597	8.585	15.861	17.136	12.398	–0.585	0.012
2024/1/25	standard	ETH2	8.597	8.588	15.861	17.067	13.066	–0.620	0.013
2024/1/25	standard	ETH3	19.743	25.539	45.902	51.810	61.634	–0.183	0.011
2024/1/25	standard	ETH3	19.750	25.546	45.924	53.107	66.031	–0.197	0.011
2024/1/25	standard	NB4	16.640	18.641	35.441	33.434	27.630	–0.361	0.009
2024/1/25	standard	P1	15.452	21.255	37.016	38.201	34.676	–0.192	0.013
2024/1/25	standard	P1	15.463	21.271	37.030	40.272	40.325	–0.172	0.012
2024/1/27	standard	P1	15.439	21.255	37.008	42.122	35.690	–0.168	0.012
2024/1/27	standard	NB4	16.652	18.664	35.405	34.591	17.858	–0.393	0.011
2024/1/27	standard	P1	15.441	21.271	37.019	41.336	33.071	–0.197	0.009
2024/1/28	standard	P1	15.451	21.260	37.021	41.004	23.654	–0.172	0.010
2024/1/28	standard	NB4	16.645	18.662	35.430	36.204	16.005	–0.378	0.010
2024/1/28	standard	P1	15.467	21.269	37.008	40.712	30.202	–0.203	0.009

2024/1/29	standard	P1	15.449	21.258	37.022	35.652	39.265	–0.175	0.012
2024/1/29	standard	NB4	16.668	18.671	35.457	43.501	20.695	–0.365	0.013
2024/1/29	standard	P1	15.457	21.268	37.020	38.552	32.003	–0.188	0.010
2025/1/21	sample	HTG-9	21.101	21.909	42.182	33.842	25.264	–0.219	0.012
2025/1/21	sample	XJG-2	20.154	21.514	40.148	42.402	23.590	–0.196	0.013
2025/1/21	sample	XJG-8	22.620	22.502	44.007	35.020	39.204	–0.206	0.008
2025/1/22	sample	HTG-9	21.109	21.896	42.196	30.151	26.964	–0.207	0.011
2025/1/22	sample	XJG-2	20.136	21.508	40.122	40.631	17.007	–0.172	0.009
2025/1/22	sample	XJG-8	22.618	22.513	44.021	32.966	31.010	–0.201	0.007
2025/1/23	sample	HTG-9	21.123	21.923	42.206	37.205	34.682	–0.234	0.010
2025/1/23	sample	XJG-2	20.115	21.519	40.110	45.238	14.130	–0.189	0.013
2025/1/23	sample	XJG-8	22.607	22.501	44.028	39.220	45.113	–0.222	0.008
2025/1/15	standard	ETH1	20.153	25.071	45.330	70.228	53.087	–0.583	0.010
2025/1/15	standard	ETH1	20.168	25.084	45.344	76.566	65.303	–0.549	0.010
2025/1/15	standard	ETH2	8.633	8.590	15.859	14.503	9.631	–0.623	0.009
2025/1/15	standard	ETH2	8.620	8.575	15.850	20.365	11.035	–0.590	0.012
2025/1/15	standard	ETH3	19.767	25.566	45.908	46.034	61.286	–0.181	0.012
2025/1/15	standard	ETH4	8.259	8.506	16.568	17.483	12.680	–0.375	0.009
2025/1/15	standard	NB4	16.660	18.651	35.451	45.630	23.602	–0.390	0.014
2025/1/15	standard	P1	15.452	21.260	37.017	33.328	29.508	–0.185	0.011
2025/1/15	standard	P1	15.458	21.255	37.026	43.529	28.233	–0.179	0.010
2025/1/21	standard	P1	15.433	21.242	37.007	35.649	39.265	–0.165	0.012
2025/1/21	standard	NB4	16.641	18.661	35.472	31.070	31.263	–0.368	0.010
2025/1/21	standard	P1	15.452	21.249	37.020	34.039	24.306	–0.179	0.008
2025/1/22	standard	P1	15.453	21.260	37.035	30.625	31.525	–0.182	0.015
2025/1/22	standard	NB4	16.640	18.659	35.450	30.281	30.475	–0.372	0.009
2025/1/22	standard	P1	15.471	21.265	37.040	39.006	25.508	–0.205	0.010
2025/1/23	standard	P1	15.462	21.260	37.032	33.595	20.561	–0.177	0.012
2025/1/23	standard	NB4	16.663	18.646	35.459	39.534	19.237	–0.381	0.012
2025/1/23	standard	P1	15.452	21.242	37.008	38.025	30.259	–0.189	0.013

REFERENCES AND NOTES

1. J. C. G. Walker, K. C. Lohmann, Why the oxygen isotopic composition of sea water changes with time. *Geophys. Res. Lett.* **16**, 323–326 (1989).

2. K. Wallmann, The geological water cycle and the evolution of marine $\delta^{18}O$ values. *Geochim. Cosmochim. Acta* **65**, 2469–2485 (2001).

3. J. B. D. Jaffrés, G. A. Shields, K. Wallmann, The oxygen isotope evolution of seawater: A critical review of a long-standing controversy and an improved geological water cycle model for the past 3.4 billion years. *Earth Sci. Rev.* **83**, 83–122 (2007).

4. J. F. Kasting, M. T. Howard, K. Wallmann, J. Veizer, G. Shields, J. Jaffrés, Paleoclimates, ocean depth, and the oxygen isotopic composition of seawater. *Earth Planet. Sci. Lett.* **252**, 82–93 (2006).

5. J. Veizer, A. Prokoph, Temperatures and oxygen isotopic composition of Phanerozoic oceans. *Earth Sci. Rev.* **146**, 92–104 (2015).

6. J. M. Ferry, B. H. Passey, C. Vasconcelos, J. M. Eiler, Formation of dolomite at 40-80 °C in the Latemar carbonate buildup, Dolomites, Italy, from clumped isotope thermometry. *Geology* **39**, 571–574 (2011).

7. P. Li, H. Zou, F. Hao, X. Yu, G. Wang, J. M. Eiler, Using clumped isotopes to determine the origin of the Middle Permian Qixia Formation dolostone, NW Sichuan Basin, China. *Mar. Pet. Geol.* **122**, 104660 (2020).

8. U. Ryb, J. M. Eiler, Oxygen isotope composition of the Phanerozoic ocean and a possible solution to the dolomite problem. *Proc. Natl. Acad. Sci. U.S.A.* **115**, 6602–6607 (2018).

9. J. Veizer, P. Bruckschen, F. Pawellek, A. Diener, O. G. Podlaha, G. A. F. Carden, T. Jasper, C. Korte, H. Strauss, K. Azmy, D. Ala, Oxygen isotope evolution of Phanerozoic seawater. *Palaeogeogr. Palaeoclimatol. Palaeoecol.* **132**, 159–172 (1997).

10. N. Galili, A. Shemesh, R. Yam, I. Brailovsky, M. Sela-adler, E. M. Schuster, C. Collom, A. Bekker, N. Planavsky, F. A. Macdonald, A. Préat, M. Rudmin, W. Trela, U. Sturesson, J. M. Heikoop, M. Aurell, J. Ramajo, I. Halevy, The geologic history of seawater oxygen isotopes from marine iron oxides. *Science* **365**, 469–473 (2019).

11. T. Isson, S. Rauzi, Oxygen isotope ensemble reveals Earth's seawater, temperature, and carbon cycle history. *Science* **383**, 666–670 (2024).

12. K. Muehlenbachs, The oxygen isotopic composition of the oceans, sediments and the seafloor. *Chem. Geol.* **145**, 263–273 (1998).

13. H. D. Holland, The oxygenation of the atmosphere and oceans. *Philos. Trans. R. Soc. B* **361**, 903–915 (2006).

14. J. M. Eiler, "Clumped-isotope" geochemistry— The study of naturally-occurring, multiply-substituted isotopologues. *Earth Planet. Sci. Lett.* **262**, 309–327 (2007).

15. S.-T. Kim, J. R. O'Neil, Equilibrium and nonequilibrium oxygen isotope effects in synthetic carbonates. *Geochim. Cosmochim. Acta* **61**, 3461–3475 (1997).

16. D. A. Stolper, J. M. Eiler, J. A. Higgins, Modeling the effects of diagenesis on carbonate clumped-isotope values in deep- and shallow-water settings. *Geochim. Cosmochim. Acta* **227**, 264–291 (2018).

17. P. Ghosh, J. Adkins, H. Affek, B. Balta, W. F. Guo, E. A. Schauble, D. Schrag, J. M. Eiler, ^{13}C-^{18}O bonds in carbonate minerals: A new kind of paleothermometer. *Geochim. Cosmochim. Acta* **70**, 1439–1456 (2006).

18. D. Bajnai, W. Guo, C. Spötl, T. B. Coplen, K. Methner, N. Löffler, E. Krsnik, E. Gischler, M. Hansen, D. Henkel, G. D. Price, J. Raddatz, D. Scholz, J. Fiebig, Dual clumped isotope thermometry resolves kinetic biases in carbonate formation temperatures. *Nat. Commun.* **11**, 4005 (2020).

19. A. J. Davies, W. Guo, M. Bernecker, M. Tagliavento, J. Raddatz, E. Gischler, S. Flögel, J. Fiebig, Dual clumped isotope thermometry of coral carbonate. *Geochim. Cosmochim. Acta* **338**, 66–78 (2022).

20. J. Fiebig, M. Daëron, M. Bernecker, W. F. Guo, G. Schneider, R. Boch, S. M. Bernasconi, J. Jautzy, M. Dietzel, Calibration of the dual clumped isotope thermometer for carbonates. *Geochim. Cosmochim. Acta* **312**, 235–256 (2021).

21. C. Lu, P. K. Swart, The application of dual clumped isotope thermometer (Δ_{47} and Δ_{48}) to the understanding of dolomite formation. *Geology* **52**, 56–60 (2024).

22. R. C. Cummins, S. Finnegan, D. A. Fike, J. M. Eiler, W. W. Fischer, Carbonate clumped isotope constraints on Silurian ocean temperature and seawater $\delta^{18}O$. *Geochim. Cosmochim. Acta* **140**, 241–258 (2014).

23. G. A. Henkes, B. H. Passey, E. L. Grossman, B. J. Shenton, T. E. Yancey, A. P. Huerta, Temperature evolution and the oxygen isotope composition of Phanerozoic oceans from carbonate clumped isotope thermometry. *Earth Planet. Sci. Lett.* **490**, 40–50 (2018).

24. S. Banerjee, P. Ghosh, Y. Banerjee, R. Riding, Oxygen isotopic composition of Paleoproterozoic seawater revealed by clumped isotope analysis of dolomite, Vempalle Formation, Cuddapah, India. *Chem. Geol.* **621**, 121356 (2023).

25. A. Liu, D. Tang, X. Shi, X. Zhou, L. Zhou, M. Shang, Y. Li, H. Fang, Mesoproterozoic oxygenated deep seawater recorded by early diagenetic carbonate concretions from the member IV of the Xiamaling Formation, North China. *Precambrian Res.* **341**, 105667 (2020).

26. G. Luo, C. Hallmann, S. Xie, X. Ruan, R. E. Summons, Comparative microbial diversity and redox environments of black shale and stromatolite facies in the Mesoproterozoic Xiamaling Formation. *Geochim. Cosmochim. Acta* **151**, 150–167 (2015).

27. S. Zhang, X. Wang, E. U. Hammarlund, H. Wang, M. M. Costa, C. J. Bjerrum, J. N. Connelly, B. Zhang, L. Bian, D. E. Canfield, Orbital forcing of climate 1.4 billion years ago. *Proc. Natl. Acad. Sci. U.S.A.* **112**, E1406–E1413 (2015).

28. W. B. Su, H. K. Li, W. D. Huff, F. R. Ettensohn, S. H. Zhang, H. Y. Zhou, Y. S. Wan, SHRIMP U-Pb dating for a K-bentonite bed in the Tieling Formation, North China. *Chin. Sci. Bull.* **55**, 3312–3323 (2010).

29. S. H. Zhang, Y. Zhao, Z. Y. Yang, Z. F. He, H. Wu, The 1.35 Ga diabase sills from the northern North China Craton: Implications for breakup of the Columbia (Nuna) supercontinent. *Earth Planet. Sci. Lett.* **288**, 588–600 (2009).

30. X. Wang, S. Zhang, Y. Ye, S. Ma, J. Su, H. Wang, D. E. Canfield, Nitrogen cycling during the Mesoproterozoic as informed by the 1400 million year old Xiamaling Formation. *Earth Sci. Rev.* **243**, 104499 (2023).

31. H. Li, S. Lu, W. Su, Z. Xiang, H. Zhou, Y. Zhang, Recent advances in the study of the Mesoproterozoic geochronology in the North China Craton. *J. Asian Earth Sci.* **72**, 216–227 (2013).

32. L. Z. Gao, C. H. Zhang, X. Y. Shi, B. Song, Z. Q. Wang, Y. M. Liu, Mesoproterozoic age for Xiamaling Formation in North China Plate indicated by zircon SHRIMP dating. *Chin. Sci. Bull.* **53**, 2665–2671 (2008).

33. S. Lu, G. Zhao, H. Wang, G. Hao, Precambrian metamorphic basement and sedimentary cover of the North China Craton: A review. *Precambrian Res.* **160**, 77–93 (2008).

34. Z. Feng, Q. Zhang, Y. Liu, L. Li, L. Jiang, J. Zhou, W. Li, Y. Ma, Reconstruction of Rodinia supercontinent: Evidence from the Erguna Block (NE China) and adjacent units in the eastern Central Asian orogenic Belt. *Precambrian Res.* **368**, 106467 (2022).

35. S. Li, X. Li, G. Wang, Y. Liu, Z. Wang, T. Wang, X. Cao, X. Guo, I. Somerville, Y. Li, J. Zhou, L. Dai, S. Jiang, H. Zhao, Y. Wang, G. Wang, S. Yu, Global Meso-Neoproterozoic plate reconstruction and formation mechanism for Precambrian basins: Constraints from three cratons in China. *Earth Sci. Rev.* **198**, 102946 (2019).

36. S.-H. Zhang, Y. Zhao, H. Ye, J.-M. Liu, Z.-C. Hu, Origin and evolution of the Bainaimiao arc belt: Implications for crustal growth in the southern Central Asian orogenic belt. *Geol. Soc. Am. Bull.* **126**, 1275–1300 (2014).

37. Z. Yi, Y. Liu, J. G. Meert, T. Wang, B. Huang, A new view of the Pangea supercontinent with an emphasis on the East Asian blocks. *Earth Planet. Sci. Lett.* **611**, 118143 (2023).

38. Y. Wang, L. Zhou, L. Zhao, Cratonic reactivation and orogeny: An example from the northern margin of the North China Craton. *Gondw. Res.* **24**, 1203–1222 (2013).

39. J. R. Kelson, K. W. Huntington, A. J. Schauer, C. Saenger, A. R. Lechler, Toward a universal carbonate clumped isotope calibration: Diverse synthesis and preparatory methods suggest a single temperature relationship. *Geochim. Cosmochim. Acta* **197**, 104–131 (2017).

40. S. V. Petersen, W. F. Defliese, C. Saenger, M. Daëron, K. W. Huntington, C. M. John, J. R. Kelson, S. M. Bernasconi, A. S. Colman, T. Kluge, G. A. Olack, A. J. Schauer, D. Bajnai, M. Bonifacie, S. F. M. Breitenbach, J. Fiebig, A. B. Fernandez, G. A. Henkes, D. Hodell, A. Katz, S. Kele, K. C. Lohmann, B. H. Passey, M. Y. Peral, D. A. Petrizzo, B. E. Rosenheim, A. Tripati, R. Venturelli, E. D. Young, I. Z. Winkelstern, Effects of improved ^{17}O correction on interlaboratory agreement in clumped isotope calibrations, estimates of mineral-specific offsets, and temperature dependence of acid digestion fractionation. *Geochem. Geophys. Geosyst.* **20**, 3495–3519 (2019).

41. N. T. Anderson, J. R. Kelson, S. Kele, M. Daëron, M. Bonifacie, J. Horita, T. J. Mackey, C. M. John, T. Kluge, P. Petschnig, A. B. Jost, K. W. Huntington, S. M. Bernasconi, K. D. Bergmann, A unified clumped isotope thermometer calibration (0.5–1,100°C) using carbonate-based standardization. *Geophys. Res. Lett.* **48**, e2020GL092069 (2021).

42. S. M. Bernasconi, M. Daëron, K. D. Bergmann, M. Bonifacie, A. N. Meckler, H. P. Affek, N. Anderson, D. Bajnai, E. Barkan, E. Beverly, D. Blamart, L. Burgener, D. Calmels, C. Chaduteau, M. Clog, B. Davidheiser-Kroll, A. Davies, F. Dux, J. Eiler, B. Elliott, A. C. Fetrow, J. Fiebig, S. Goldberg, M. Hermoso, K. W. Huntington, E. Hyland, M. Ingalls, M. Jaggi, C. M. John, A. B. Jost, S. Katz, J. Kelson, T. Kluge, I. J. Kocken, A. Laskar, T. J. Leutert, D. Liang, J. Lucarelli, T. J. Mackey, X. Mangenot, N. Meinicke, S. E. Modestou, I.

A. Müller, S. Murray, A. Neary, N. Packard, B. H. Passey, E. Pelletier, S. Petersen, A. Piasecki, A. Schauer, K. E. Snell, P. K. Swart, A. Tripati, D. Upadhyay, T. Vennemann, I. Winkelstern, D. Yarian, N. Yoshida, N. Zhang, M. Ziegler, InterCarb: A community effort to improve interlaboratory standardization of the carbonate clumped isotope thermometer using carbonate standards. *Geochem. Geophys. Geosyst.* **22**, e2020GC009588 (2021).

43. J. Fiebig, D. Bajnai, N. Löffler, K. Methner, E. Krsnik, A. Mulch, S. Hofmann, Combined high-precision Δ_{48} and Δ_{47} analysis of carbonates. *Chem. Geol.* **522**, 186–191 (2019).

44. P. K. Swart, C. J. Lu, E. W. Moore, M. E. Smith, S. T. Murray, P. T. Staudigel, A calibration equation between Δ_{48} values of carbonate and temperature. *Rapid Commun. Mass Spectrom.* **35**, e9147 (2021).

45. J. Veizer, D. Ala, K. Azmy, P. Bruckschen, D. Buhl, F. Bruhn, G. A. F. Carden, A. Diener, S. Ebneth, Y. Godderis, T. Jasper, C. Korte, F. Pawellek, O. G. Podlaha, H. Strauss, $^{87}Sr/^{86}Sr$, $\delta^{13}C$ and $\delta^{18}O$ evolution of Phanerozoic seawater. *Chem. Geol.* **161**, 59–88 (1999).

46. R. L. Folk, Practical petrographic classification of limestones. *AAPG Bull.* **43**, 1–38 (1959).

47. L. C. Kah, T. W. Lyons, J. T. Chesley, Geochemistry of a 1.2 Ga carbonate-evaporite succession, northern Baffin and Bylot Islands: Implications for Mesoproterozoic marine evolution. *Precambrian Res.* **111**, 203–234 (2001).

48. Z.-D. Bao, H.-C. Ji, Y. Wang, Z.-F. Li, T. Liang, B. Niu, M.-Y. Wei, K. Lu, Y.-Q. Shi, H. Zhang, P. Wan, Z.-L. Li, Z.-B. Yang, R. Liu, C.-X. Liu, X.-L. Zhong, X.-Q. Guo, P.-F. Xiang, Z.-X. Cai, S.-C. Zhang, The primary dolostone in the Meso-Neoproterozoic: Cases study on platforms in China. *J. Palaeogeogr.* **11**, 151–172 (2022).

49. M. Kunzmann, S. Schmid, T. N. Blaikie, G. P. Halverson, Facies analysis, sequence stratigraphy, and carbon isotope chemostratigraphy of a classic Zn-Pb host succession: The Proterozoic middle McArthur Group, McArthur Basin, Australia. *Ore Geol. Rev.* **106**, 150–175 (2019).

50. Y. Guo, W. Deng, G. Wei, Kinetic effects during the experimental transition of aragonite to calcite in aqueous solution: Insights from clumped and oxygen isotope signatures. *Geochim. Cosmochim. Acta* **248**, 210–230 (2019).

51. W. Guo, Kinetic clumped isotope fractionation in the DIC-H_2O-CO_2 system: Patterns, controls, and implications. *Geochim. Cosmochim. Acta* **268**, 230–257 (2020).

52. B. H. Passey, G. A. Henkes, Carbonate clumped isotope bond reordering and geospeedometry. *Earth Planet. Sci. Lett.* **351**, 223–236 (2012).

53. D. A. Stolper, J. M. Eiler, The kinetics of solid-state isotope-exchange reactions for clumped isotopes: A study of inorganic calcites and apatites from natural and experimental samples. *Am. J. Sci.* **315**, 363–411 (2015).

54. G. A. Henkes, B. H. Passey, E. L. Grossman, B. J. Shenton, A. P. Huerta, T. E. Yancey, Temperature limits for preservation of primary calcite clumped isotope paleotemperatures. *Geochim. Cosmochim. Acta* **139**, 362–382 (2014).

55. J. J. Sweeney, A. K. Burnham, Evaluation of a simple model of vitrinite reflectance based on chemical kinetics. *AAPG Bull.* **74**, 1559–1570 (1990).

56. L. P. Knauth, S. Epstein, Hydrogen and oxygen isotope ratios in nodular and bedded cherts. *Geochim. Cosmochim. Acta* **40**, 1095–1108 (1976).

57. F. Robert, M. Chaussidon, A palaeotemperature curve for the Precambrian oceans based on silicon isotopes in cherts. *Nature* **443**, 969–972 (2006).

58. S.-T. Kim, J. R. O'Neil, C. Hillaire-Marcel, A. Mucci, Oxygen isotope fractionation between synthetic aragonite and water: Influence of temperature and Mg^{2+} concentration. *Geochim. Cosmochim. Acta* **71**, 4704–4715 (2007).

59. S. R. Thorrold, S. E. Campana, C. M. Jones, P. K. Swart, Factors determining $\delta^{13}C$ and $\delta^{18}O$ fractionation in aragonitic otoliths of marine fish. *Geochim. Cosmochim. Acta* **61**, 2909–2919 (1997).

60. C. Lécuyer, A. Hutzler, R. Amiot, V. Daux, D. Grosheny, O. Otero, F. Martineau, F. Fourel, V. Balter, B. Reynard, Carbon and oxygen isotope fractionations between aragonite and calcite of shells from modern molluscs. *Chem. Geol.* **332**, 92–101 (2012).

61. L. P. Knauth, D. R. Lowe, High Archean climatic temperature inferred from oxygen isotope geochemistry of cherts in the 3.5 Ga Swaziland Supergroup, South Africa. *Geol. Soc. Am. Bull.* **115**, 566–580 (2003).

62. E. J. Judd, J. E. Tierney, D. J. Lunt, I. P. Montanez, B. T. Huber, S. L. Wing, P. J. Valdes, A 485-million-year history of Earth's surface temperature. *Science* **385**, eadk3705 (2024).

63. A. J. Kaufman, S. Xiao, High CO_2 levels in the Proterozoic atmosphere estimated from analyses of individual microfossils. *Nature* **425**, 279–282 (2003).

64. S. Zhang, X. Wang, H. Wang, C. J. Bjerrum, E. U. Hammarlund, M. M. Costa, J. N. Connelly, B. Zhang, J. Su, D. E. Canfield, Sufficient oxygen for animal respiration 1,400 million years ago. *Proc. Natl. Acad. Sci. U.S.A.* **113**, 1731–1736 (2016).

65. K. Muehlenbachs, R. N. Clayton, Oxygen isotope composition of the oceanic crust and its bearing on seawater. *J. Geophys. Res.* **81**, 4365–4369 (1976).

66. G. C. Zhao, P. A. Cawood, S. A. Wilde, M. Sun, Review of global 2.1–1.8 Ga orogens: Implications for a pre-Rodinia supercontinent. *Earth Sci. Rev.* **59**, 125–162 (2002).

67. C. Herzberg, K. Condie, J. Korenaga, Thermal history of the Earth and its petrological expression. *Earth Planet. Sci. Lett.* **292**, 79–88 (2010).

68. Y. Zou, R. N. Mitchell, X. Chu, M. Brown, J. L. Jiang, Q. L. Li, L. Zhao, M. G. Zhao, Surface evolution during the mid-Proterozoic stalled by mantle warming under Columbia–Rodinia. *Earth Planet. Sci. Lett.* **607**, 118055 (2023).

69. M. Tang, X. Chu, J. Hao, B. Shen, Orogenic quiescence in Earth's middle age. *Science* **371**, 728–731 (2021).

70. Q. Luo, L. Zhang, N. Zhong, J. Wu, F. Goodarzi, H. Sanei, C. B. Skovsted, V. Suchý, M. Li, X. Ye, W. Cao, A. Liu, X. Min, Y. Pan, L. Yao, J. Wu, Thermal evolution behavior of the organic matter and a ray of light on the origin of vitrinite-like maceral in the Mesoproterozoic and Lower Cambrian black shales: Insights from artificial maturation. *Int. J. Coal Geol.* **244**, 103813 (2021).

71. H. Jacob, Classification, structure, genesis and practical importance of natural oil bitumen (“migrabitumen”). *Int. J. Coal Geol.* **11**, 65–79 (1989).

72. K. W. Huntington, J. M. Eiler, H. P. Affek, W. F. Guo, S. M. Bonifacie, L. Y. Yeung, N. Thiagarajan, B. Passey, A. Tripati, M. Daëron, R. Came, Methods and limitations of ‘clumped’ CO_2 isotope (Δ_{47}) analysis by gas-source isotope ratio mass spectrometry. *J. Mass Spectrom.* **44**, 1318–1329 (2009).

73. R. A. Eagle, E. A. Schauble, A. K. Tripati, T. Tütken, R. C. Hulbert, J. M. Eiler, Body temperatures of modern and extinct vertebrates from ^{13}C-^{18}O bond abundances in bioapatite. *Proc. Natl. Acad. Sci. U.S.A.* **107**, 10377–10382 (2010).

74. S. M. Bernasconi, I. A. Müller, K. D. Bergmann, S. F. M. Breitenbach, A. Fernandez, D. A. Hodell, M. Jaggi, A. N. Meckler, I. Millan, M. Ziegler, Reducing uncertainties in carbonate clumped isotope analysis through consistent carbonate-based standardization. *Geochem. Geophys. Geosyst.* **19**, 2895–2914 (2018).

75. A. N. Meckler, M. Ziegler, M. I. Millan, S. F. M. Breitenbach, S. M. Bernasconi, Long-term performance of the Kiel carbonate device with a new correction scheme for clumped isotope measurements. *Rapid Commun. Mass Spectrom.* **28**, 1705–1715 (2014).

76. S. M. Bernasconi, B. Hu, U. Wacker, J. Fiebig, S. F. M. Breitenbach, T. Rutz, Background effects on Faraday collectors in gas-source mass spectrometry and implications for clumped isotope measurements. *Rapid Commun. Mass Spectrom.* **27**, 603–1612 (2013).

77. C. M. John, D. Bowen, Community software for challenging isotope analysis: First applications of ‘Easotope’ to clumped isotopes. *Rapid Commun. Mass Spectrom.* **30**, 2285–2300 (2016).

78. X. Wang, S. Zhang, H. Wang, C. J. Bjerrum, E. U. Hammarlund, E. R. Haxen, J. Su, Y. Wang, D. E. Canfield, Oxygen, climate and the chemical evolution of a 1400 million year old tropical marine setting. *Am. J. Sci.* **317**, 861–900 (2017).

79. T. G. Wang, N. N. Zhang, C. J. Wang, Y. X. Zhu, Y. Liu, D. F. Song, Source beds and oil entrapment-alteration histories of fossil-oil-reservoirs in the Xiamaling Formation Basal Sandstone, Jibei Depression. *Pet. Sci. Bull.* **01**, 24–37 (2016).

80. J. J. W. Rogers, M. Santosh, Supercontinents in Earth history. *Gondw. Res.* **6**, 357–368 (2003).
